# Surgical or percutaneous coronary revascularization for heart failure: an *in silico* model using routinely collected health data to emulate a clinical trial

**DOI:** 10.1093/eurheartj/ehac670

**Published:** 2022-11-25

**Authors:** Suraj Pathak, Florence Y Lai, Joanne Miksza, Mark C Petrie, Marius Roman, Sarah Murray, Jeremy Dearling, Divaka Perera, Gavin J Murphy

**Affiliations:** Cardiovascular Research Centre, University of Leicester, Glenfield Hospital, Groby Road, Leicester, LE3 9QP, UK; Cardiovascular Research Centre, University of Leicester, Glenfield Hospital, Groby Road, Leicester, LE3 9QP, UK; Cardiovascular Research Centre, University of Leicester, Glenfield Hospital, Groby Road, Leicester, LE3 9QP, UK; School of Cardiovascular & Metabolic Health BHF GCRC, Glasgow, G12 8TA, UK; Cardiovascular Research Centre, University of Leicester, Glenfield Hospital, Groby Road, Leicester, LE3 9QP, UK; National Cardiac Surgery Patient and Public Involvement (PPI) Group, University of Leicester, Glenfield Hospital, Groby Road, Leicester, LE3 9QP, UK; National Cardiac Surgery Patient and Public Involvement (PPI) Group, University of Leicester, Glenfield Hospital, Groby Road, Leicester, LE3 9QP, UK; Cardiovascular Division, Rayne Institute, Lambeth Wing, Westminster Bridge Road, London, SE1 7EH, UK; Cardiovascular Research Centre, University of Leicester, Glenfield Hospital, Groby Road, Leicester, LE3 9QP, UK

**Keywords:** Clinical trial emulation, Hospital episode statistics, Coronary artery disease, Revascularization, Coronary artery bypass grafting, Percutaneous coronary intervention, Trial feasibility

## Abstract

**Aims:**

The choice of revascularization with coronary artery bypass grafting (CABG) vs. percutaneous coronary intervention (PCI) in people with ischaemic left ventricular dysfunction is not guided by high-quality evidence.

**Methods and results:**

A trial of CABG vs. PCI in people with heart failure (HF) was modelled *in silico* using routinely collected healthcare data. The *in silico* trial cohort was selected by matching the target trial cohort, identified from Hospital Episode Statistics in England, with individual patient data from the Surgical Treatment for Ischemic Heart Failure (STICH) trial. Allocation to CABG vs. complex PCI demonstrated random variation across administrative regions in England and was a valid statistical instrument. The primary outcome was 5-year all-cause mortality or cardiovascular hospitalization. Instrumental variable analysis (IVA) was used for the primary analysis. Results were expressed as average treatment effects (ATEs) with 95% confidence intervals (CIs). The target population included 13 519 HF patients undergoing CABG or complex PCI between April 2009 and March 2015. After matching, the emulated trial cohort included 2046 patients. The unadjusted primary outcome rate was 51.1% in the CABG group and 70.0% in the PCI group. IVA of the emulated cohort showed that CABG was associated with a lower risk of the primary outcome (ATE −16.2%, 95% CI −20.6% to −11.8%), with comparable estimates in the unmatched target population (ATE −15.5%, 95% CI −17.5% to −13.5%).

**Conclusion:**

In people with HF, *in silico* modelling suggests that CABG is associated with fewer deaths or cardiovascular hospitalizations at 5 years vs. complex PCI. A pragmatic clinical trial is needed to test this hypothesis and this trial would be feasible.

## Structured Graphical Abstract

**Structured Graphical Abstract:**
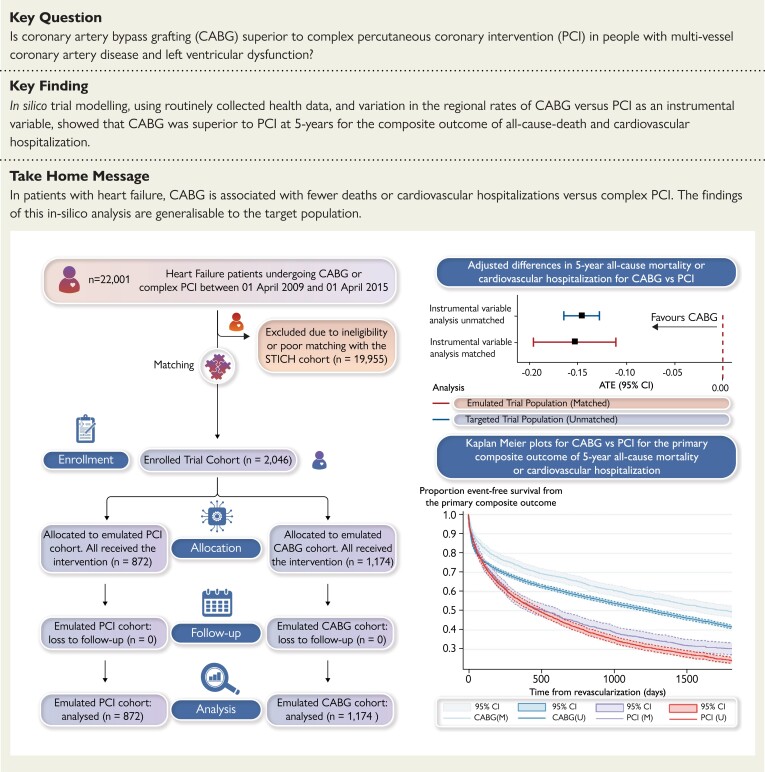
*In silico* trial modelling, using variation in the regional rates of CABG vs. PCI as an instrumental variable, suggests that CABG is superior to PCI at 5 years for the primary composite outcome of all-cause-death or cardiovascular hospitalization. M, matched—emulated trial cohort; U, unmatched—targeted trial cohort; CABG, coronary artery bypass graft; PCI, percutaneous coronary intervention; CI, confidence interval.

**See the editorial comment for this article ‘Revascularization in ischaemic cardiomyopathy: how to interpret current evidence’, by B.J. Gersh and D. De Mets, https://doi.org/10.1093/eurheartj/ehac794.**

## Introduction

Heart failure (HF) affects 1%–2% of the population, causes severe symptoms, high rates of mortality, frequent hospitalizations, and costs the UK National Health Service (NHS) £2bn per year.^1^ Coronary artery disease (CAD) is the most common cause of HF with reduced ejection fraction (HFrEF).^2^ The Surgical Treatment for Ischemic Heart Failure (STICH) trial failed to show treatment benefit in patients aged >60 years or those enrolled in Western Europe or North America.^3^ Moreover, advances in pharmacological therapy since the STICH trial, mean that there remains significant uncertainty as to whether revascularization is superior to medical therapy, and if so, which revascularization strategy is optimal. Currently, patients with HF and CAD routinely undergo revascularization with coronary artery bypass grafting (CABG) or percutaneous coronary interventions (PCIs), despite there being limited evidence that it improves symptoms of HF or prognosis. The choice of the revascularization strategy is not guided by high quality evidence, because most randomized controlled trials (RCTs) comparing the effectiveness of CABG vs. PCI excluded patients with HF.^4,5^ In the Synergy between PCI with Taxus and Cardiac Surgery (SYNTAX) trial, for example, only 1.8% of participants had left ventricular ejection fraction (LVEF) < 30%.^6^ Therefore, the evidence from these studies is not generalizable to people with HF, who have different relative risks and benefits for both CABG and PCI, when compared with people without HF.^7^ No RCT has compared CABG vs. PCI in this patient population. This means that these high-risk patients are routinely managed by Heart Teams in the absence of evidence.

Randomized controlled trials are challenging in high-risk groups. The need for extended timelines and high costs arise from lengthy governance processes, the requirement for feasibility studies, and failure to meet recruitment targets. One way to abbreviate this process is to model trials *in silico* using existing large-scaled routinely collected healthcare data. *In silico* trials are computational methods that use routinely collected healthcare data, pre-specified eligibility criteria, allocations to exposures of interest (treatments), measurement of outcomes of interest, along with advanced analytics, to emulate clinical trials. The results do not replace the need for clinical trials but can provide information on feasibility, outcome event rates, treatment effects sizes, optimal design of future clinical trials, and likely generalizability of these trials to the patient population as a whole.

The aims of the current study were to follow a structured process to model clinical trials *in silico* using Health Episodes Statistics (HES) in England linked to the national death registry, and to apply this framework to test whether CABG is superior to complex PCI in people with multi-vessel CAD and HF.

## Methods

### Design

The outline of the target trial is illustrated in *Table 1*. Briefly, the design was as follows: patients: adults with a hospitalization for HF who had undergone revascularization (PCI or CABG). Intervention: CABG. Comparator: complex PCI. Outcome (primary): composite of all-cause mortality or cardiovascular hospitalization at 5 years.

**Table 1:** Target trial and emulated protocols for a superiority trial of coronary artery bypass grafting vs. percutaneous coronary intervention in the UK

	Target trial	Emulated trial
Trial design	Parallel	Parallel
Blinding	Open label	Open label
Setting	30 cardiac centres in the UK	All patients undergoing NHS care in England
Eligibility criteria of participants	**Inclusion criteria:** Adults (>18) with HF – LVEF ≤40% (any modality). Severe coronary artery disease. Significant amount of myocardium at risk because of coronary artery disease [BCIS myocardial jeopardy score >6 on recent coronary angiogram (<6 months)]. Heart team believes that revascularization can be achieved by both PCI or CABG. Informed consent.	**Inclusion criteria:** HF diagnosis in any hospital admissions within 2 years prior to the index revascularization. Severe coronary artery disease defined by patients who had undergone CABG or complex PCI (revascularization).
	**Exclusion criteria:** Perioperative cardiogenic shock. Decompensated HF <48 h prior to randomization. Myocardial infarction within 30 days of randomization. Previous valvular heart disease requiring surgical repair/replacement. Known high bleeding risk. Pregnancy.	**Exclusion criteria:** Cardiogenic shock in the preceding 7 days. Decompensated HF, defined by: (i) LVAD insertion, (ii) CVC or arterial line insertion, (iii) ventilation or (iv) HF, within 2 days prior to revascularization. MI within 30 days prior to the index intervention date. Valve surgery within 2 years prior to the index date. Known high bleeding risk (2 year lookback) defined by: chronic kidney disease (CKD), stroke, transfusion, bleeding diathesis, liver disease with portal hypertension, malignancy, brain arteriovenous malformation. Maternal admission, either in the preceding, or the year following, the index intervention.
		**Not feasible to phenotype the following criteria due to lack of available data:** LVEF ≤40% (any modality). BCIS myocardial jeopardy score >6 on recent (<6 months) coronary angiogram. Heart team believes that revascularization can be achieved by either PCI or CABG. Informed consent.
Comparative populations	Intervention: PCI Control: CABG	Patients undergoing CABG (OPCS4 K40–K46) were compared with patients undergoing high-risk complex PCI procedures. Complex PCI was defined as either: (i) A PCI involving multiple coronary arteries (OPCS4 K492), or (ii) A PCI with deployment of 3 or more stents (OPCS4 K752, K754), or (iii) A staged PCI where the patient underwent more than one PCI intervention (OPCS4 K49, K50, K75) within 90 days. Index date of intervention was defined as the date of revascularization, whether CABG or PCI. For patients undergoing a staged PCI, index date is defined as the date of the first PCI.
Treatment allocation	Randomization in 1:1 ratio—computer generated	Randomization was emulated by harnessing the naturally occurring regional variation in treatment practices across hospitals. Using the regional surgical rate as an instrument variable, we conducted instrument variable analysis which controlled for known and unknown confounders to estimate treatment effects.
Recruitment	Patients with HF will be identified in cardiac catheterization day wards and in heart team meetings over 4 years.	Patients with a prior diagnosis of HF hospitalized between 2009 and 2015 undergoing CABG or complex PCI. Index episodes and all hospital admissions within 2 years prior to the index episode were used to establish patient characteristics and eligibility.
Follow up	Follow-up following revascularization (CABG or PCI) until 5 years.	Follow-up after the index intervention (CABG or complex PCI) for a minimum of 5 years.
Primary outcome	Composite of all-cause mortality or cardiovascular hospitalization at 5 years.	Composite of All-cause mortality and Cardiovascular hospitalizations (ICD I00–I99) identified based on primary diagnosis at 5 years. Sensitivity analysis to be conducted using both primary and secondary diagnosis.
Secondary outcome	(1) All-cause mortality at 5 years. (2) Cardiovascular hospitalization at 5 years. (3) Cardiovascular-cause mortality at 5 years. (4) Myocardial infarction at 5 years. (5) Stroke at 5 years (6) Other major adverse events e.g. HF hospitalization, venous thromboembolism at 5 years. (7) Quality of life measured using the EQ-5D-5L at 5 years. (8) Quality-of-life adjusted days-alive-out-of-hospital at 5 years.	(1) All-cause mortality at 5 years. (2) Cardiovascular hospitalization at 5 years. (3) Cardiovascular-cause mortality at 5 years. (4) Hospitalizations for myocardial infarction at 5 years. (5) Hospitalizations for stroke at 5 years. (6) Hospitalizations for other major adverse events: HF hospitalization at 5 years. (7) Hospitalizations for other major adverse events: venous thromboembolism at 5 years.
		**Not feasible to phenotype the following outcomes due to lack of available data:** (1) Quality of life measured using the EQ-5D-5L at 5 years. (2) Quality-of-life adjusted days-alive-out-of-hospital at 5 years.
Statistical analysis	Both intention to treat and per protocol analyses will be reported. Primary outcome will be analysed using the Kaplan–Meier method with event or censoring times estimated from the date of randomization. Differences in outcomes between treatment arms will be assessed using the log-rank test.	As we expect imbalance across our patient groups, IVA will be used to estimate the treatment effects with the instrumental variable being the regional surgical rates. IVA can potentially adjust for observed and unobserved confounders. Sensitivity analysis used alternative analytic models for estimating adjusted treatment effects including (i) multivariable regression adjustment, (ii) propensity score matching, and (iii) meta-learning models.

LVEF, left ventricular ejection fraction; CVC, central venous catheter; LVAD, left ventricular assist device; CABG, coronary artery bypass graft; PCI, percutaneous coronary intervention; BCIS, British Cardiac Intervention Society; CKD, chronic kidney disease; OPCS-4, classification of interventions and procedures; ICD10, international classification of diseases; EQ-5D-5L, EuroQol-5 dimension-5 level; IVA, instrumental variable analysis; HF, heart failure.

**Figure 1:**
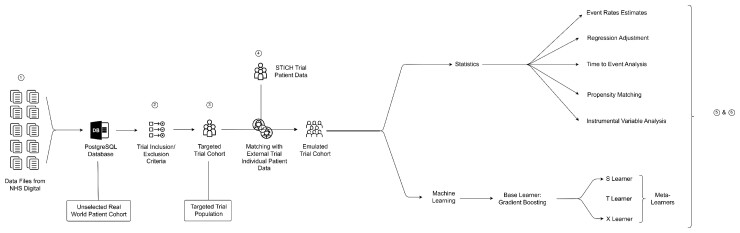
Trial modelling framework. ① Data cleaning, harmonization and linking. ② An *in silico* trial protocol is developed as a best approximation of the target trial protocol given the limitations of the routinely collected HES data. ③ Phenotyping algorithms developed using ICD 10 and OPCS-4 codes are applied to the Health Episodes Statistics database to shortlist a targeted patient cohort. ④ The targeted patient cohort is matched to an existing trial, targeting the same population (STICH), based on key patient baseline characteristics, to derive the emulated trial cohort, which approximates a likely trial cohort. ⑤ Statistical and machine learning methods are applied to the emulated trial population to provide information on trial feasibility, outcome event rates and treatment effects sizes.

The protocol for the target trial was co-developed by patients and clinicians as part of the National Cardiac Surgery Clinical Trial Initiative.^8,9^ The working group consisted of 46 stakeholders, of which 26 members were service users. In total 12 investigator meetings were held between 2020 and 2021 along with 2 dedicated Patient and Public Involvement workshops. All patient members had personal experience of cardiovascular disease either as patients or carers and came from a diverse range of backgrounds.^9^ The target trial protocol was used to develop an emulated trial protocol, which included all the key design features of the target trial, and gave the best approximation of the trial protocol given the limitations and constraints of the observational data used (*Table 1*). To model the trial *in silico*, first, we implemented the eligibility criteria, identified the intervention and comparator populations (target trial population), and mapped out the outcome event rates using HES data, as specified in the emulated trial protocol. To mimic an actual trial population for analysis, as an estimate of the generalizability of a trial to the overall UK population, the emulated trial cohort was selected from the target trial population, by matching with individual patient data from the STICH trial cohort^5^ (*Figure 1*).

Due to the non-randomized nature of the data, we used statistical methods that mitigated the effect of confounding on treatment effect estimates, a key limitation of observational analyses. Specifically, the primary analysis used the random variation in the proportions of patients treated with complex PCI vs. CABG across regions in England as a statistical instrument to assess treatment effects. We used instrumental variable analysis (IVA) to estimate the treatment effects of CABG vs. complex PCI for the primary outcome. Sensitivity analyses were used to test the reproducibility of these estimates across alternative analytical approaches and outcome definitions. Finally, we outlined the design of a future pragmatic clinical trial that would confirm or refute our *in silico* findings.

The study received appropriate governance approvals from the University of Leicester Research Ethics Committee, NHS Digital, and the National Institutes of Health (NIH).

### Data sources

Data sources included (i) UK HES, routinely collected healthcare data for all hospital admissions at NHS hospitals in England linked to the National Death Registry and (ii) individual participant data from the STICH trial; the only high quality RCT to date that evaluated revascularization in the target population, from the NIH.^3^

### Setting

All NHS hospitals in England (HES).^3^

### Patient cohort

Patients hospitalized for HF (ICD10 I50) in England between 2009 and 2015, who underwent revascularization (CABG or PCI) within the subsequent 2 years, were identified from the HES Admitted Patient Care dataset. Left ventricular ejection fraction is not available in HES and therefore we could not identify a HFrEF subset. Individual patient data from the STICH trial, detailing baseline status, interventions, and outcomes were acquired from the NIH.

### Case ascertainment and outcome definitions

Phenotyping algorithms for defining study cohorts, inclusion and exclusion criteria, patient comorbidities and trial outcomes of interests are provided in *Table 1* and Supplementary material online, *Tables S1–S4*. We defined index episodes as the hospital episodes in which revascularization (CABG or complex PCI) was performed. Coronary artery bypass grafting was defined by OPCS4 codes K40–K46. To model a trial in patients with multivessel disease, where either surgery or PCI may be indicated, we included only patients undergoing complex PCI in the PCI arm. Non-complex PCI was excluded as we judged that surgery would be less likely to be indicated in these patients. Complex PCI was defined by either of the following conditions: (i) a single PCI involving multiple coronary arteries (OPCS4 K492) or deployment of three or more stents (OPCS4 K752, K754), or (ii) a staged PCI where the patient underwent more than one PCI intervention (OPCS4 K49, K50, K75) within 90 days.

This definition would include PCI procedures described in contemporary trials of multivessel revascularization.^6,10^

Patient demographics including age, sex, ethnicity, and index of multiple deprivation (IMD) were extracted from the index episode. Diagnoses and procedures performed in all hospital episodes within 2 years prior to the index episodes were used to establish patients’ medical history and eligibility to the trial. Diagnoses recorded in hospital episodes following the index episodes were used to map out the outcome events. Linkage to the UK National Death Registry (Office for National Statistics) was used to record deaths outside hospitals.

The primary outcome was a composite of 5-year cardiovascular hospitalization or all-cause mortality that has a low risk of detection bias (see Supplementary material online, *Table S4*). Secondary outcomes included: cardiovascular mortality, myocardial infarction (MI), stroke, HF hospitalization, cardiovascular hospitalization, venous thromboembolism (VTE) (see Supplementary material online, *Table S4*). Patients were followed up for a minimum of 5 years.

Hospitalization outcomes were identified using the primary diagnosis recorded in HES. Death outcomes were ascertained from HES hospital and the National Death Registry. Myocardial infarction can either be an indication for revascularization, or a complication following the intervention, we therefore began follow-up for this outcome from the next admission following the index episode. Follow-up for cardiovascular rehospitalization began the day after the intervention for patients undergoing CABG or a complex PCI intervention. For staged PCI, follow-up for cardiovascular hospitalization began from the first PCI episode within the 90-day window.

### Matching the targeted trial cohort to the STICH trial cohort

Patients participating in clinical trials are healthier and carry fewer risk factors than the target trial population due to the voluntary basis of participation.^11,12^ To account for this potential bias in our model, we matched our targeted trial population obtained from HES, to an existing trial cohort, targeting the same high-risk patient population, based on important prespecified baseline characteristics. This was used to derive the emulated patient cohort, which mimics an actual trial population. In this study, we matched our HES-derived HF patient cohort to the individual patient data of the STICH trial^5^ using logistic propensity score matching. The model included key clinically important covariates available across both cohorts including age, gender, and comorbidities including diabetes, hypertension, lipidaemia, chronic kidney disease (CKD), previous MI or peripheral vascular disease.

### Statistical analysis

Continuous variables are expressed as medians with interquartile ranges, and categorical variables as numbers with corresponding percentages. Comparisons between groups were performed using Mann–Whitney *U* test or Pearson’s *χ*^2^ test as appropriate. Two-sided *P*-values were used for all analyses. The analysis of the primary outcome in the target trial would be based on time-to-first-event analyses using the Kaplan–Meier method. We expected imbalance across our patient groups, IVA was therefore used as the primary analysis method to estimate treatment effects for the *in silico* trial.

Instrumental variable analysis is a two-stage regression which is often used to account for both observed and unobserved confounders. The first stage regression is between the instrumental variable and treatment selection. The second stage regression is between the outcome and the predicted probability of treatment selection, using the model built in the first stage. A good instrumental variable should strongly correlate with the treatment allocation and is not associated with any patient characteristics (observed or unobserved). A Recursive Bivariate Probit (*RBiProbit*, STATA) model was used to model the 5-year composite outcome of all-cause mortality or cardiovascular hospitalization.^13^ At both stages, the models controlled for the following prespecified covariates: age, gender, ethnicity, IMD and comorbidities (diabetes, hypertension, lipidaemia, CKD, previous stroke, previous MI). The instrumental variable model estimated average treatment effects (ATEs) with 95% confidence intervals (CIs), interpreted as absolute risk differences.

In this study, regional variation (derived from the outward postcode) in physician preference for the use of CABG or PCI, was used as the instrument variable. This instrument performs like a natural randomization of patients to regional treatment groups, as the instrument directly dictates treatment allocation, but is independent of patient characteristics, as a result, it can be used to remove the effects of hidden bias.

A funnel plot (*FunnelplotR*, R-package) was used to investigate variations in regional surgical rates by plotting standardized surgical ratios against the expected number of CABG surgeries in each geographical region. A logistics model adjusting for age, gender, comorbidities, ethnicity and IMD, was used to estimate the expected number of surgeries in each region.

### Subgroup analyses

To explore any potential heterogeneity in treatment effects, a subgroup analysis was completed for all patients in our cohort, stratified by prespecified patient factors. Treatment effects were reported as ATEs with 95% CI, from a Probit regression model (*teffects*, STATA).

### Sensitivity analysis

Alternative analytical models to estimate ATEs, including multivariable regression adjustment, propensity score matching, and meta-learning methods, were implemented as sensitivity analysis.

For regression adjustment, we used a multivariable Probit regression model (*teffects*, STATA) which controlled for the following prespecified covariates: age, gender, ethnicity, IMD and comorbidities (diabetes, hypertension, lipidaemia, CKD, previous stroke, previous MI). Estimates were expressed as ATEs with 95% CI, interpreted as absolute risk differences.

For propensity score matching, propensity scores were estimated using a Probit regression model (*PSMatch2*, STATA) with the binary dependent variable being treatment allocation and covariates being age, gender, ethnicity, IMD and comorbidities (diabetes, hypertension, lipidaemia, CKD, previous stroke, previous MI). Variables were selected to be included in the propensity scoring model based on clinical expertise. Patients with missing data were excluded from the analysis. Patients were sampled without replacement. A nearest neighbour algorithm with a calliper of 0.01 was used to optimize the matches between the CABG and PCI groups. Standardized mean differences were used to check balance after propensity score matching.^14^ All unmatched individuals were excluded from the analysis. Estimates were expressed as ATEs with 95% CI.

The final class of estimators implemented were a series of meta-learning models (S-learners, T-learners and X-learners).^15^ Meta-learners break estimation of within-person treatment effects into (at least) two, separate steps: (i) models are trained to estimate outcomes for intervention and control groups, and then (ii) for every observed outcome, a counterfactual, alternative outcome is generated from a fitted model, this is then used to estimate the within-person treatment effect, and then generalized to provide the ATE across the cohort. Meta-learning approaches have several key advantages over traditional methods in causal inference, all of which have been described in the supplementary material, along with their individual algorithms (see Supplementary material online, *Figures S1–S4*). In this analysis, extreme gradient boosting (XGBoost) was used as the base learner for each of the three separate meta-learning algorithms. Rigorous benchmarking analyses have consistently demonstrated that XGBoost models offer the best combination of prediction performance and processing time and was therefore used in this analysis.^11,12,16–18^

In HES, each episode records one primary diagnosis and up to 19 secondary diagnoses. For the primary analysis, the outcome measures were defined using primary diagnoses only. As a sensitivity analysis, we also repeated all analyses and reported the treatment effects using all available primary and secondary diagnoses to define outcomes.

We used R (V4.2) and STATA (V17) to run the statistical analyses and Python (V3.10) to run the machine learning models. Data were managed using PostgreSQL (V13) and DataGrip (V2022.2).

### Trial design

Sample sizes for a future clinical trial to confirm or refute our findings were estimated based on time-to-event analysis, for a superiority trial, comparing CABG (intervention) to complex PCI (control) (*ARTSURV*, STATA).^19^ Event rates along with survival probabilities for the anticipated study duration were obtained from our HES analysis.

## Results

### Patient cohort

**Figure 2:**
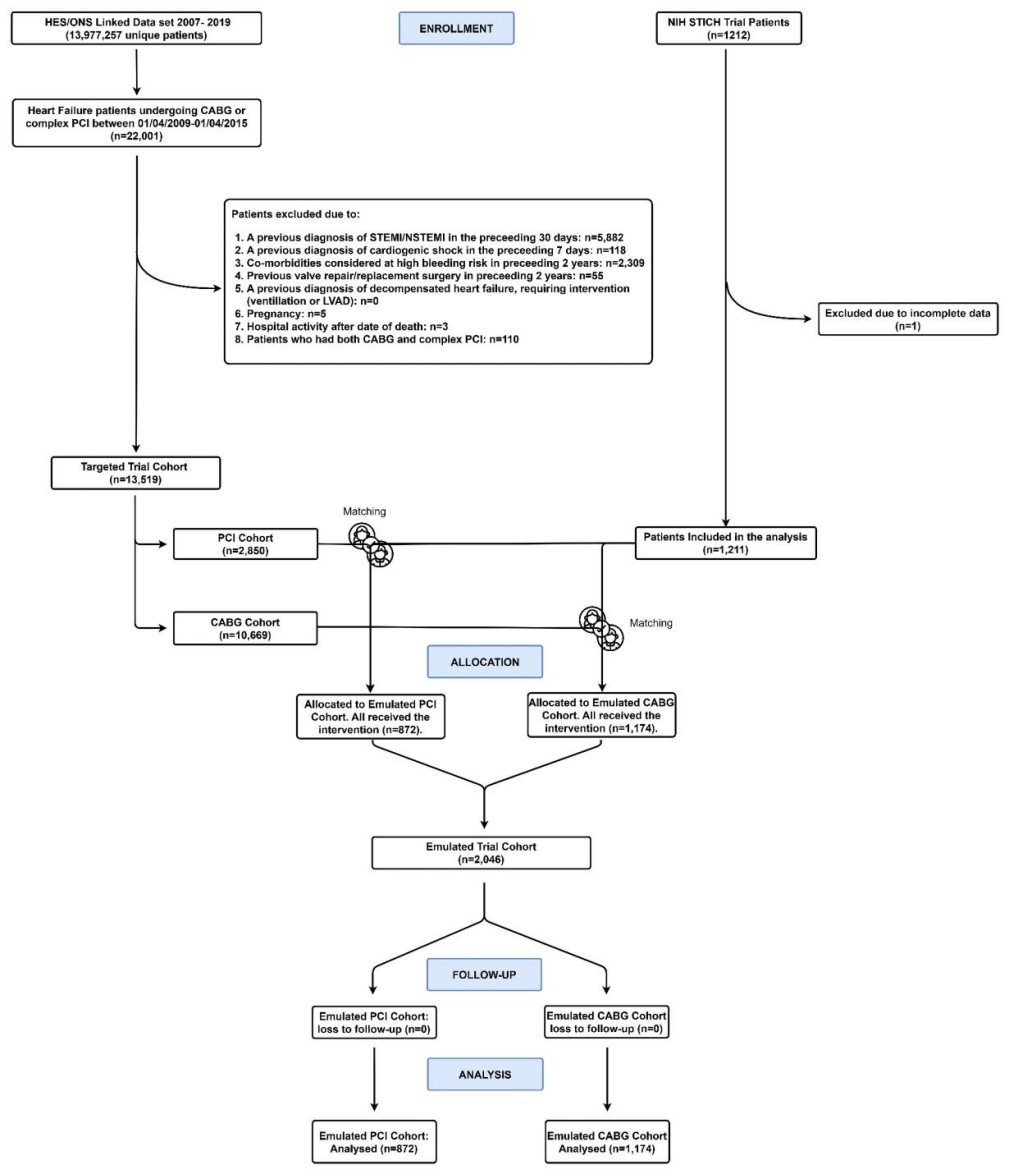
Modified CONSORT diagram describing how our cohorts were defined and the flow of patients in our analyses once the trial-modelling framework had been applied. HES, Hospital Episode Statistics; ONS, Office for National Statistics; NIH, National Institutes of Health; CABG, coronary artery bypass graft; CONSORT, consolidated standards of reporting trials; PCI, percutaneous coronary intervention; NSTEMI, non-ST elevated myocardial infarction; STEMI, ST-elevated myocardial infarction; LVAD, left ventricular assist device.

Between 1 April 2009 and 1 April 2015, 13 519 patients with HF undergoing revascularization with CABG or complex PCI met the target trial eligibility criteria and were analysed as the target trial cohort (*Figure 2*). There were 10 669 patients undergoing CABG compared with 2850 patients undergoing complex PCI. Patients undergoing complex PCI were older, living in areas with higher relative deprivation (IMD), with increased prevalence of diabetes, previous MI and CKD (*Table 2*). The Hospital Frailty Risk Score (HFRS) and Charlson Comorbidity Index (CCI)^6–9^ were greater in the population undergoing complex PCI (*Table 2*).

**Table 2:** Comparison of patient demographics and comorbidities across the STICH trial cohort, targeted trial cohort and emulated trial cohort

		STICH trial cohort	Matched HES (emulated trial cohort)	Unmatched HES (targeted trial cohort)	Unmatched cohort—targeted trial cohort			CABG	Matched cohort—emulated trial cohort	
					CABG	Complex PCI	*P*-value		Complex PCI	*P*-value
*N*		1211	2046	13 519	10 669	2850		1174	872	
Age, years, median (IQR)^a^		60.0 (54.0, 67.0)	61.0 (54.0, 69.0)	71.0 (63.0, 77.0)	71.0 (63.0, 77.0)	72.0 (64.0, 79.0)	<0.001	60.0 (53.0, 68.0)	63.0 (55.0, 70.0)	<0.001
Ethnicity^a^	Asian	—	165 (8.1%)	898 (6.6%)	651 (6.1%)	247 (8.7%)	<0.001	94 (8.0%)	71 (8.1%)	0.36
	Black	—	34 (1.7%)	113 (0.8%)	79 (0.7%)	34 (1.2%)		23 (2.0%)	11 (1.3%)	
	Mixed/other	—	42 (2.1%)	209 (1.5%)	170 (1.6%)	39 (1.4%)		27 (2.3%)	15 (1.7%)	
	White	—	1658 (81.0%)	11 249 (83.2%)	8932 (83.7%)	2317 (81.3%)		938 (79.9%)	720 (82.6%)	
	NA	—	147 (7.2%)	1050 (7.8%)	837 (7.8%)	213 (7.5%)		92 (7.8%)	55 (6.3%)	
Sex^a^	Male	1063 (87.8%)	1754 (85.7%)	10 220 (75.6%)	8151 (76.4%)	2069 (72.6%)	<0.001	1014 (86.4%)	740 (84.9%)	0.33
	Female	148 (12.2%)	292 (14.3%)	3299 (24.4%)	2518 (23.6%)	781 (27.4%)		160 (13.6%)	132 (15.1%)	
IMD, median (IQR)^b^		—	21.8 (12.1, 36.7)	18.1 (10.5, 30.7)	17.9 (10.3, 30.1)	19.4 (11.0, 32.5)	<0.001	21.1 (12.1, 35.7)	22.8 (12.1, 37.6)	0.24
Diabetes		477 (39.4%)	859 (42.0%)	5069 (37.5%)	3857 (36.2%)	1212 (42.5%)	<0.001	469 (39.9%)	390 (44.7%)	0.03
Hypertension		728 (60.1%)	1371 (67.0%)	10 662 (78.9%)	8503 (79.7%)	2159 (75.8%)	<0.001	778 (66.3%)	593 (68.0%)	0.41
Lipidaemia		729 (60.3%)	1241 (60.7%)	8312 (61.5%)	6896 (64.6%)	1416 (49.7%)	<0.001	716 (61.0%)	525 (60.2%)	0.72
CKD		94 (7.8%)	168 (8.2%)	2843 (21.0%)	2173 (20.4%)	670 (23.5%)	<0.001	80 (6.8%)	88 (10.1%)	0.008
CVA		92 (7.6%)	22 (1.1%)	289 (2.1%)	278 (2.6%)	11 (0.4%)	<0.001	17 (1.4%)	5 (0.6%)	0.058
MI		933 (77.0%)	1465 (71.6%)	4228 (31.3%)	3165 (29.7%)	1063 (37.3%)	<0.001	902 (76.8%)	563 (64.6%)	<0.001
PVD		184 (15.2%)	280 (13.7%)	1559 (11.5%)	1228 (11.5%)	331 (11.6%)	0.88	152 (12.9%)	128 (14.7%)	0.26
HFRS, median (IQR)		—	7.8 (3.1, 16.8)	10.4 (4.5, 20.1)	10.0 (4.3, 19.5)	12.0 (5.4, 22.2)	<0.001	6.6 (2.7, 15.6)	9.8 (3.9, 18.4)	<0.001
CCI, median (IQR)		—	6.0 (4.0, 11.0)	7.0 (4.0, 11.0)	6.0 (3.0, 10.0)	8.0 (5.0, 13.0)	<0.001	6.0 (3.0, 10.0)	8.0 (4.0, 13.0)	<0.001

Charlson Comorbidity Index (CCI) predicts 10-year survival in patients with multiple comorbidities (https://www.mdcalc.com/calc/3917/charlsoncomorbidity-index-cci) it is used to estimate disease burden in the different cohorts.

CABG, coronary artery bypass graft; PCI, percutaneous coronary intervention; CVA, cerebrovascular accident; CKD, chronic kidney disease; PVD, peripheral vascular disease; HF, heart failure; MI, myocardial infarction; CCI, Charlson Comorbidity Index; IMD, index of multiple deprivation; HFRS, Hospital Frailty Risk Score; IQR, interquartile range.

a No missing data for both the targeted trial and matched cohorts.

b 1.4% of data missing in the targeted trial cohort, 1.3% of data missing in the emulated trial cohort. Patients with missing data were removed from any analysis using IMD as a covariate in the model.

### Matching

Propensity score matching with logistic regression was used to match the HES-derived cohort (*n* = 13 519) with the STICH trial cohort (*n* = 1211). The CABG and complex PCI patient cohorts were matched separately with the STICH cohort. In total, 2046 patients (1174 CABG and 872 PCI patients) were matched with the STICH trial participants and included in the final analysis as the emulated trial cohort. All covariates achieved a standardized mean difference of <10% following matching (see Supplementary material online, *Figures S5* and *S6*, Supplementary material online, *Tables S5* and *S6*). The baseline characteristics of the emulated trial cohort closely resembled the STICH cohort, but were significantly younger, with fewer long-term conditions than the unmatched HES cohort (*Table 2*).

After matching with STICH, the baseline characteristics between the CABG and the PCI groups within the matched cohort were more balanced (*Table 2*), although there remained differences between the two groups. Patients undergoing CABG were younger, with lower prevalence of CKD and diabetes, but a larger proportion having a history of previous MI, when compared with patients undergoing complex PCI (*Table 2*). Both the HFRS and CCI^6–9^ remained greater in the population undergoing complex PCI (*Table 2*).

### Variation in regional surgical rates

**Figure 3:**
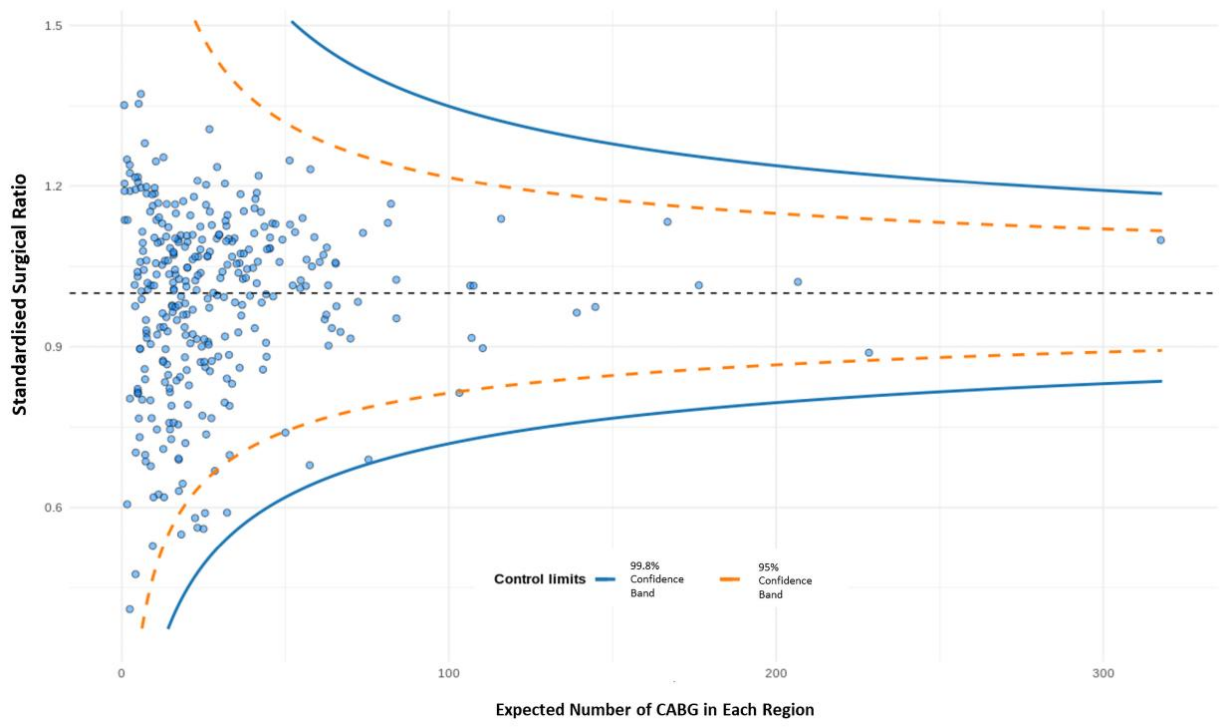
Funnel plot showing regional variation in surgical rates among revascularized heart failure patients in England. A total of 320 administrative regions in England plotted. CABG, coronary artery bypass graft; PCI, percutaneous coronary intervention.

Regional CABG rates among revascularized HF patients (CABG and complex PCI) ranged from 33% to 100% across England (mean: 77.6%, standard deviation: 11.3%). After adjusting for patient characteristics, evidence of variation in the regional surgical rates remained (*Figure 3*). The first stage *F*-statistic of 194.45 (*P* < 0.001) was greater than the Stock–Yogo critical value of 16.38, at the 10% level. This was consistent with the Anderson canonical correlations test (*P* < 0.001), which demonstrated that this instrument was strongly correlated with treatment allocation. We then analysed the baseline characteristics of the entire cohort split by quartiles of the instrument (see Supplementary material online, *Table S7*). A number of features remained well balanced across the quartiles. Although, covariates including diabetes and ethnicity showed some variation. These analyses supported the use of the regional variation in physician preference for the use of CABG or PCI as a valid instrumental variable.

### Primary outcome—matched cohort

**Figure 4:**
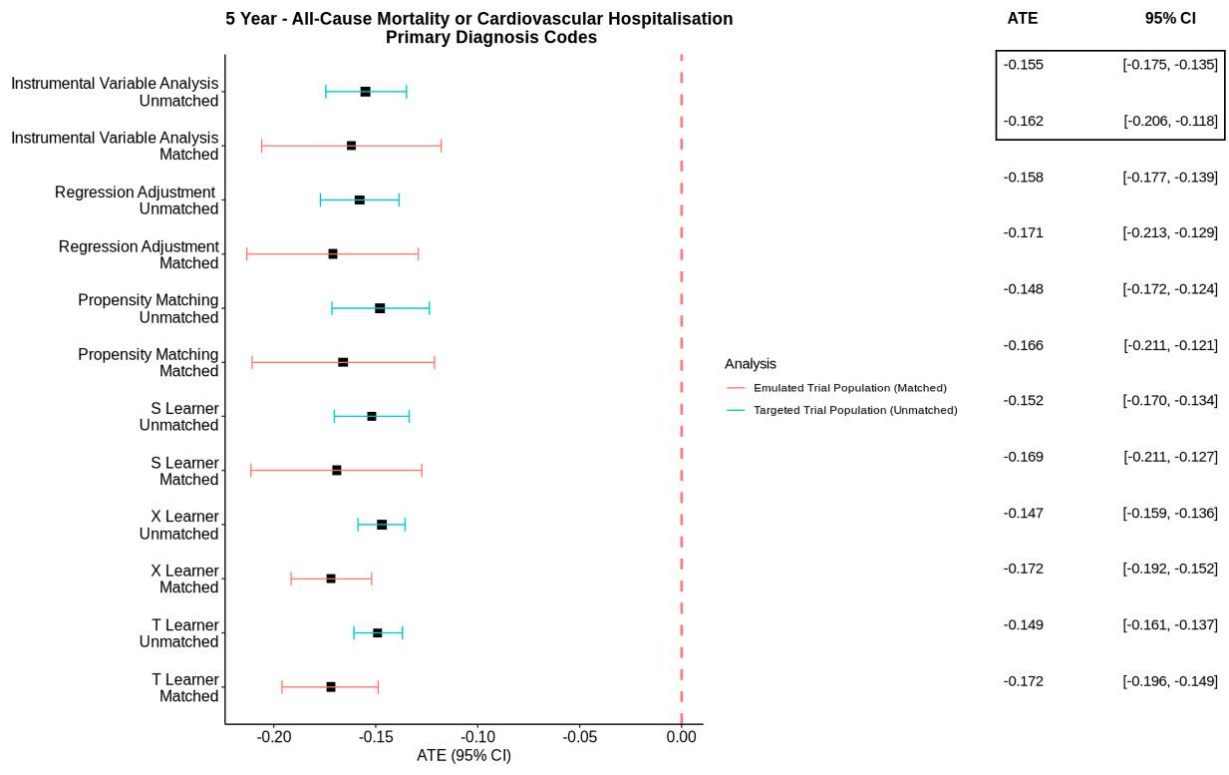
Average treatment effects with 95% confidence interval for the primary composite outcome of all-cause mortality or cardiovascular hospitalization at 5 years for both matched and unmatched cohorts. Only primary diagnosis codes were used. M, matched—emulated trial cohort; U, unmatched—targeted trial cohort; CABG, coronary artery bypass graft; PCI, percutaneous coronary intervention; 95% CI, 95% confidence interval; PD, primary diagnosis codes; ATE, average treatment effects.

In the emulated trial cohort (matched, *n* = 2046), patients undergoing CABG had lower rates of mortality (CABG 18.9%, PCI 31.8%) and cardiovascular hospitalization (CABG 42.2%, PCI 60.6%) at 5 years when compared with PCI (*Table 3*). The corresponding 5-year event rates for the composite primary outcome was 51.1% for CABG and 70.0% for PCI, with an absolute risk difference of 18.9%. With IVA, the ATE was −16.2% (95% CI −20.6% to −11.8%) (*Figure 4*).

**Table 3:** Crude event rates at 5 years for the matched and unmatched cohorts—primary diagnosis codes

	Matched—emulated trial cohort			Unmatched—targeted trial cohort		
	CABG	PCI	*P*-value	CABG	PCI	*P*-value
No of patients	1174	872		10 669	2850	
All-cause mortality	222 (18.9%)	277 (31.8%)	<0.001	3046 (28.6%)	1187 (41.6%)	<0.001
Cardiovascular mortality	130 (11.1%)	181 (20.8%)	<0.001	1842 (17.3%)	710 (24.9%)	<0.001
Myocardial infarction	59 (5.0%)	138 (15.8%)	<0.001	448 (4.2%)	434 (15.2%)	<0.001
Stroke	65 (5.5%)	33 (3.8%)	0.066	636 (6.0%)	145 (5.1%)	0.076
Heart failure hospitalization	163 (13.9%)	203 (23.3%)	<0.001	1902 (17.8%)	766 (26.9%)	<0.001
Cardiovascular hospitalization	495 (42.2%)	528 (60.6%)	<0.001	4725 (44.3%)	1758 (61.7%)	<0.001
Venous thromboembolism	10 (0.9%)	10 (1.1%)	0.50	80 (0.7%)	39 (1.4%)	0.002
Composite of all-cause mortality or cardiovascular hospitalization	600 (51.1%)	610 (70.0%)	<0.001	6267 (58.7%)	2164 (75.9%)	<0.001

CABG, coronary artery bypass graft; PCI, percutaneous coronary intervention.

### Primary outcome—unmatched cohort

**Figure 5:**
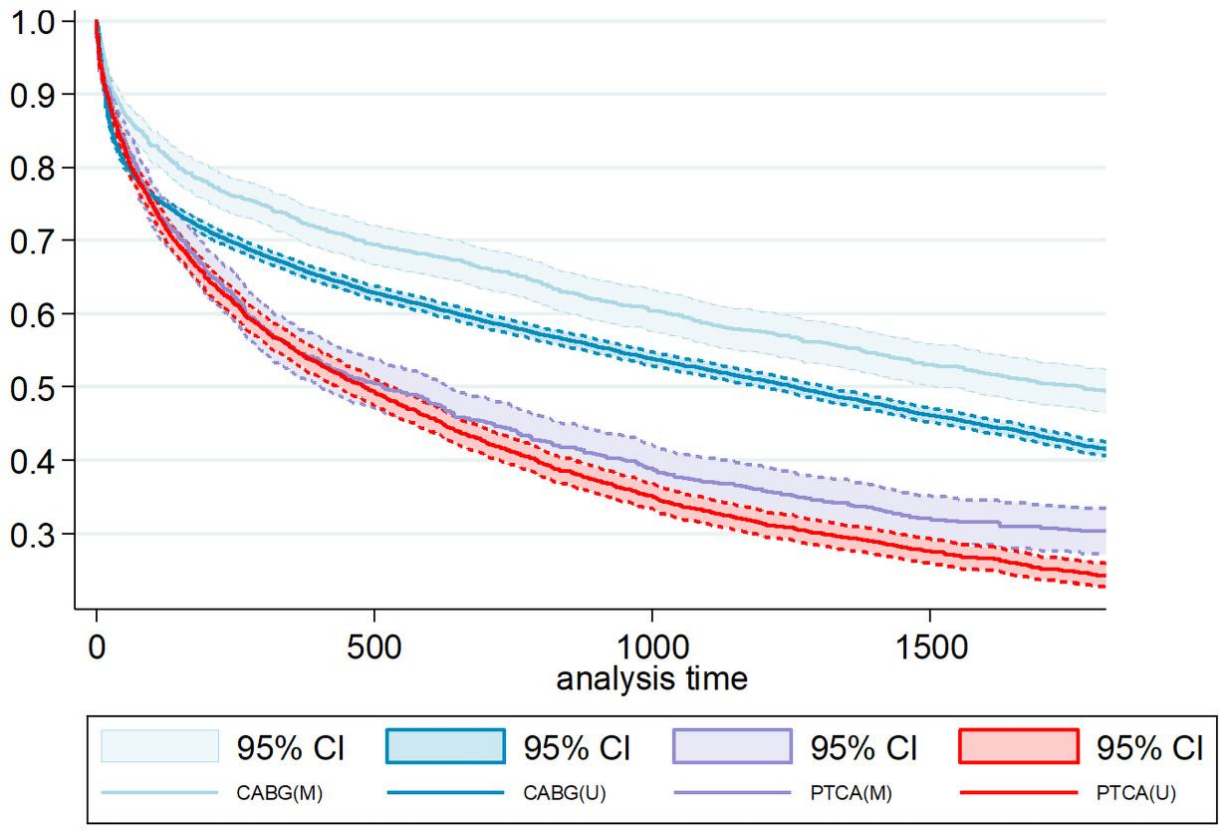
Kaplan–Meier plots for coronary artery bypass grafting vs. percutaneous coronary intervention in the matched and unmatched cohorts for the primary composite outcome of all-cause mortality or cardiovascular hospitalization. M, matched—emulated trial cohort; U, unmatched—targeted trial cohort; CABG, coronary artery bypass graft; PCI, percutaneous coronary intervention; 95% CI, 95% confidence interval; PD, primary diagnosis codes.

Unadjusted 5-year composite primary outcome rates in the target trial cohort (unmatched, *n* = 13 519) were 58.7% for CABG and 75.9% for PCI, with an absolute risk difference of 17.2% (*Table 3*). The Kaplan–Meier plots demonstrated a statistically significant survival benefit in the CABG cohort (log rank *P* < 0.001) (*Figure 5*). With IVA, the ATE at 5-year follow-up was −15.5% (95% CI −17.5% to −13.5%, *Figure 4*).

### Secondary outcomes

Adjusted analysis in the matched cohort demonstrated that patients undergoing CABG had significantly lower rates for cardiovascular hospitalization (ATE −16.7%, 95% CI −21.3% to −12.1%), all-cause mortality (ATE −8.0%, 95% CI −13.1% to −2.9%), cardiovascular mortality (ATE −7%, 95% CI −10.3% to −3.7%), HF hospitalization (ATE −7.6%, 95% CI −11.2% to −4%) and MI (ATE −11.9%, 95% CI −15.0% to −8.9%) at 5 years, when compared with complex PCI. There was no statistically significant difference in 5-year stroke (ATE −1.9%, 95% CI −0.1% to 3.8%) or 5-year VTE (ATE −0.6%, 95% CI −1.5% to 0.4%) (see Supplementary material online, *Figures S7–S13*).

### Sensitivity analysis—matched cohort

Using alternative analytical models including multivariable regression adjustment (ATE −17.1%, 95% CI −21.3% to −12.9%), propensity score matching (ATE −16.6%, 95% CI −21.1% to −12.1%, Supplementary material online, *Figures S14* and *S15* and Supplementary material online, *Tables S8–S11*) and meta-learners (S-learners: ATE −16.9%, 95% CI −21.1% to −12.7%, T-learners: ATE −17.2%, 95% CI −19.6% to −14.9%, X-learners: ATE −17.2%, 95% CI −19.2% to −15.2%), on the matched cohort, demonstrated consistent treatment effects for the primary outcome, confirming the validity of all estimates from the primary analysis (*Figure 4*). All analytical models in the primary analysis also demonstrated greater treatment effects being observed in the matched cohort compared with that in the unmatched cohort (*Figure 4*).

When the phenotyping algorithms were broadened to include both primary and secondary diagnosis codes, both the direction, magnitude, and precision of the treatment effect sizes remained consistent with the primary analysis (*Table 4*, Supplementary material online, *Figures S16–S22*). When visually inspecting the forest plots, it was clear that using both the primary and secondary diagnoses resulted in a reduction in the estimated treatment effect for both the composite outcome of all-cause mortality or cardiovascular hospitalization (see Supplementary material online, *Figure S16*), and cardiovascular hospitalization (see Supplementary material online, *Figure S17*). In contrast, there was an increase in the estimated treatment effect for cardiovascular mortality (see Supplementary material online, *Figure S18*) and HF hospitalization (see Supplementary material online, *Figure S19*). No significant impact was observed for MI, stroke or VTE (see Supplementary material online, *Figures S20–S22*).

**Table 4:** Crude event rates at 5 years for the matched and unmatched cohorts—primary and secondary diagnosis codes

	Matched—emulated trial cohort			Unmatched—targeted trial cohort		
	CABG	PCI	*P*-value	CABG	PCI	*P*-value
No of patients	1174	872		10 669	2850	
All-cause mortality	222 (18.9%)	277 (31.8%)	<0.001	3046 (28.6%)	1187 (41.6%)	<0.001
Cardiovascular mortality	192 (16.4%)	257 (29.5%)	<0.001	2642 (24.8%)	1062 (37.3%)	<0.001
Myocardial infarction	81 (6.9%)	146 (16.7%)	<0.001	613 (5.7%)	464 (16.3%)	<0.001
Stroke	85 (7.2%)	61 (7.0%)	0.83	860 (8.1%)	235 (8.2%)	0.75
Heart failure hospitalization	445 (37.9%)	476 (54.6)	<0.001	4789 (44.9%)	1765 (61.9%)	<0.001
Cardiovascular hospitalization	817 (69.6%)	711 (81.5%)	<0.001	7581 (71.1%)	2331 (81.8%)	<0.001
Venous thromboembolism	16 (1.4%)	19 (2.2%)	0.16	170 (1.6%)	76 (2.7%)	<0.001
Composite of all-cause mortality or cardiovascular hospitalization	879 (74.9%)	748 (85.8%)	<0.001	8530 (80.0%)	2539 (89.1%)	<0.001

CABG, coronary artery bypass graft; PCI, percutaneous coronary intervention.

### Subgroup analyses

Average treatment effects were evaluated for each patient subgroup and found to be generally consistent for all groups evaluated (see Supplementary material online, *Figure S23*). All high-risk patient subgroups had non-significant interaction terms. Patients with either a previous history of CKD or stroke tended to have reduced benefit when undergoing revascularization with CABG when compared with PCI, although these differences failed to reach statistical significance.

### Sample size for the target trial

To explore the feasibility of delivering a randomized clinical trial to test the superiority of CABG over PCI in patients with ischaemic cardiomyopathy, we estimated a sample size based on a time-to-event analysis^19^ for a range of important treatment effects (see Supplementary material online, *Table S12*) with the following assumptions: (i) a primary outcome with an event rate of 70% at 5 years in the PCI cohort, as shown by our analysis; (ii) 4-year accrual with equal recruitment; (iii) a total study period of 7 years, resulting in a median follow-up of 5-years; (iv) an expected crossover rate of 5%; and (v) zero attrition due to the use of HES data for the primary outcome. To detect a hazard ratio (HR) for CABG vs. PCI of 0.7, which falls within the range of treatment effects (0.63–0.74) observed in previous pragmatic RCTs evaluating revascularization strategies that have changed clinical practice,^6,20,21^ a total of 370 events or 592 participants (*n* = 296 in each arm) would be required. Given that over 2000 patients meet the eligibility criteria for the target trial protocol per year in England (over 13 000 eligible patients identified over 6 years), a superiority trial testing our hypothesis should be considered feasible.

## Discussion

We presented a structured, transparent, and reproducible process to model clinical trials *in silico* using routinely collected health data. The results of our *in silico* trial suggests that the risk of the death or cardiovascular hospitalization at 5 years was significantly lower amongst HF patients who underwent CABG, when compared with those who underwent PCI (*Structured Graphical Abstract*). The primary analytical approach used IVA in an attempt to adjust for hidden bias. When the analysis was performed in the emulated trial cohort matched with STICH trial participants, who were younger with fewer comorbidities than the unselected English patient population, treatment effects were increased. Treatment effects measured in a future clinical trial may therefore overestimate the treatment effect of CABG vs. PCI in the real world, but the differences will be small. These observations were consistent across multiple sensitivity analyses and secondary outcomes. Subgroup analyses demonstrated consistent treatment effects across all high-risk patient groups. Finally, the results demonstrated the feasibility of a pragmatic clinical trial to test a superiority hypothesis in English cardiac centres.

### Strengths

The strengths of this analytical approach include, first, the use of a comprehensive national dataset reducing the likelihood of sampling bias, and increasing the likely generalizability of the results. Second, HES data have a primary diagnostic coding accuracy of 96% from 2002 onwards, whilst operative coding has been found to be 97% accurate, reducing the likelihood of detection or attrition bias.^22,23^ Third, our data demonstrated that regional surgical rates can serve as an effective instrumental variable. The variable was normally distributed, highly correlated with treatment allocation, and largely independent of patient characteristics, increasing the likelihood that the analysis has adjusted for unmeasured confounders. Fourth, linkage of HES and death registry data provided long-term follow-up at 5 years. These data are typically not available from feasibility studies or published data. The follow-up period can also be modified as required to assist with the design of a future trial. Fifth, by matching our analysis to STICH trial participants, we were able to confirm the generalizability of any trial findings to the population as a whole. Sixth, this approach can be used to model any future clinical trial where the interventions and diagnoses are coded in HES.

### Limitations

Important limitations of the approach include, first, in this study we did not have access to data on LVEF and therefore do not know whether our analysis applies to the subset of patients with HFrEF; the phenotype enrolled in the STICH trial. Second, the analyses utilized observational data intended for administrative use. Therefore, despite controlling for both known and unknown confounders, any associations cannot be considered causal. Third, bias by indication in this setting would strongly favour CABG which is performed in people considered fit to withstand surgery, whereas PCI is often used in people who are not as fit. This bias is evident from our data and favours longer-term outcomes in the CABG group. We used IVA in an attempt to mitigate this important source of bias. Previous observational cohort studies comparing long-term outcomes for CABG vs. PCI in ischaemic cardiomyopathy which used statistical methods that do not adjust for unmeasured confounders demonstrated much larger treatment effects in favour of CABG, than reported here, providing further support.^24^ However, we noted treatment effect estimates similar to the IVA analysis across sensitivity analyses that did not adjust for unmeasured confounders including propensity matching and logistic regression. This is suggestive of residual confounding in our own analysis, which is not unexpected as there are still residual differences in measured covariates across regions. Fourth, imaging data, prescribing data (renin-angiotensin-aldosterone system blockade, beta-blocker, mineralocorticoid receptor antagonist), non-pharmacological therapy use, laboratory data, requirement for social care, and quality of life data are not available in HES, which limited our ability to phenotype patient cohorts at baseline, determine completeness of revascularization, or to validate outcomes. Neither could we model patient preference for one treatment vs. another. Fifth, matching was limited to the STICH cohort. This trial recruited participants between 2002 and 2007, prior to publication of the SYNTAX trial^6^ and other pivotal trials of myocardial revascularization strategies. Subsequent developments in diagnosis and treatment strategies may change the characteristics of patients who are recruited to a contemporary clinical trial. In mitigation, STICH was the only published high quality trial of revascularization in ischaemic cardiomyopathy available at the time of the analysis. Sixth, we could not reproduce the judgement that ‘the heart team believes that revascularization can be achieved by either PCI or CABG’, *in silico*. In mitigation however, we approximated this criterion by including those who either had complex PCI or CABG, as surgical interventions are less likely to be indicated in patients who required non-complex PCI. In addition, we addressed this by clear reporting of both the proposed and emulated trial protocols, as well as the trial modelling process, so that this is reproducible, to enable qualified interpretation of the data. Seventh, this study used data from NHS hospitals across England. Therefore, the results from this study can only be generalized patients who are treated in the English public sector healthcare environment. Finally, the patients included in this study underwent their respective intervention between 2009 and 2015. As a result, improvements in clinical care due to the evolution of clinical practice and introduction of new innovative approaches in PCI may not have been captured in this analysis.

### Clinical importance

There is uncertainty about the best revascularization strategy for patients with HF and CAD, whether or not LVEF is reduced.^25–27^ In STICH, the HR for the primary analysis of the primary endpoint (all-cause mortality) was 0.86 (95% CI 0.72–1.04; *P* = 0.12) but with extended follow-up was 0.84 (95% CI 0.73–0.97; *P* = 0.02) in favour of CABG.^3,28^ Interestingly, guideline recommendations on revascularization for patients with HFrEF are rather discordant, although there is general agreement that CABG should be preferred over PCI.^25,26^ Heart failure guidelines, written mainly by physicians and nurses, give a rather weak recommendation (Class IIb) with a surprisingly low level of evidence (Level C) for CABG.^25^ In contrast, guidelines on myocardial revascularization, written mainly by surgeons and interventional cardiologists, give a strong recommendation (Class I) for CABG with a moderate level of evidence (Level B).^26^

In the REVIVED-BCIS2 trial, PCI was non-inferior to best medical therapy alone in people with ischaemic cardiomyopathy, who were considered ineligible for surgery (HR 0.99; 95% CI 0.78–1.27; *P* = 0.96).^10^ Although not immediately generalizable to our target trial population, the REVIVED-BCIS2 results point towards limited effectiveness of PCI in people with HF.

No trial has compared CABG vs. PCI in this population. As shown in the current analysis, this knowledge gap is associated with unwarranted variation in practice that is associated with variation in outcomes. Here we show that a superiority RCT of CABG vs. PCI is feasible. The international STICH consortium is currently conducting nation specific trials of CABG vs. PCI in HF across seven countries (NCT05427370, NCT05329285). These trials will not deliver results until the end of the decade. In the absence of high quality RCT evidence, and the limitations of our analysis notwithstanding, the results of the current analysis suggest that CABG is the treatment of choice in ischaemic cardiomyopathy.

## Conclusions

*In silico* trial modelling, using IVA, indicates that the risk of all-cause death or cardiovascular hospitalization at 5 years, was significantly lower amongst HF patients who underwent CABG, when compared with those who underwent PCI. The treatment estimates are robust across multiple sensitivity analyses. A pragmatic clinical trial is needed to test this hypothesis and our analysis showed that this trial would be feasible.

## Authors’ contributions

G.J.M.—Clinical Expertise, Conception, Trial Protocol Development, Phenotyping, Supervision, Manuscript Review. D.P.—Clinical Expertise, Trial Protocol Development, Phenotyping, Manuscript Review. M.C.P. —Clinical Expertise, Trial Protocol Development, Phenotyping, Manuscript Review. F.Y.L.—Conception, Phenotyping, Supervision, Manuscript Review, Statistical Expertise. S.P.—Conception, Phenotyping, Data analysis, Manuscript Drafting, Manuscript Review. J.M.—Statistical Expertise, Manuscript Review. M.R.—Clinical Expertise, Phenotyping, Manuscript Review. S.M.—Co-led trial protocol development, Manuscript Review. J.D.—Co-led trial protocol development, Manuscript Review.

## Supplementary Material

ehac670_Supplementary_Data

## Data Availability

The data underlying this article were provided by NHS Digital under licence. The data that support the findings of this study are available from the corresponding author upon reasonable request. Raw data may be shared with permission of NHS Digital.
