# Supplementary material for: Surgical or percutaneous coronary revascularization for heart failure: an in silico model using routinely collected health data to emulate a clinical trial
Source: Eur Heart J. 2022 Nov 25;44(5):351–64. doi: 10.1093/eurheartj/ehac670 (PMC9890210; doi:10.1093/eurheartj/ehac670)
Supplement: ehac670_Supplementary_Data [file ehac670_supplementary_data.docx]

**Surgical or Percutaneous Coronary Revascularisation for Heart Failure: An *In Silico* Model using Routinely Collected Health Data to Emulate a Clinical Trial.**

**Supplementary Figures and Tables**

**1.0 Phenotyping**


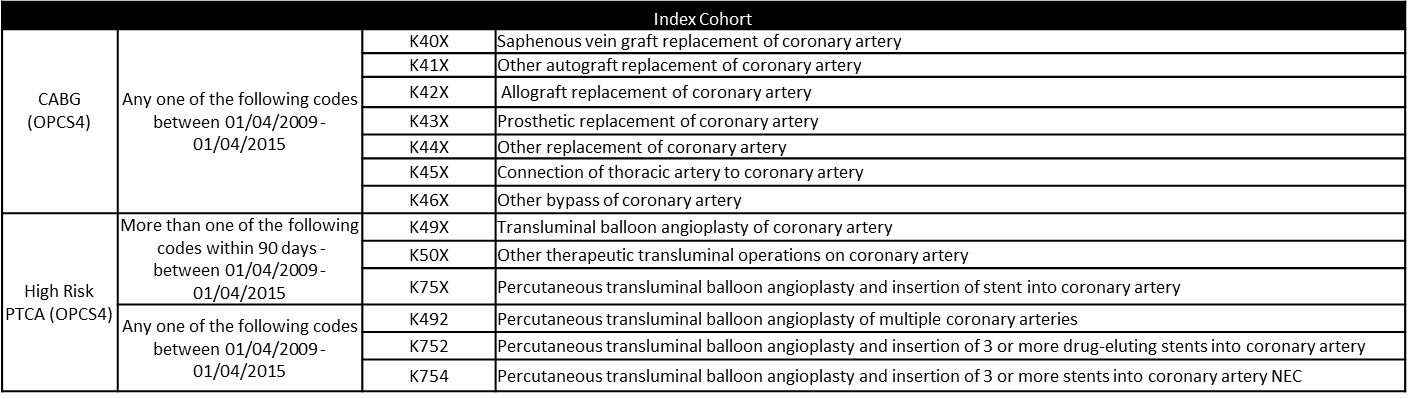


Complex PCI (OPCS-4)

**eTable 1**: OPCS-4 codes used to phenotype the complex PCI and CABG cohorts


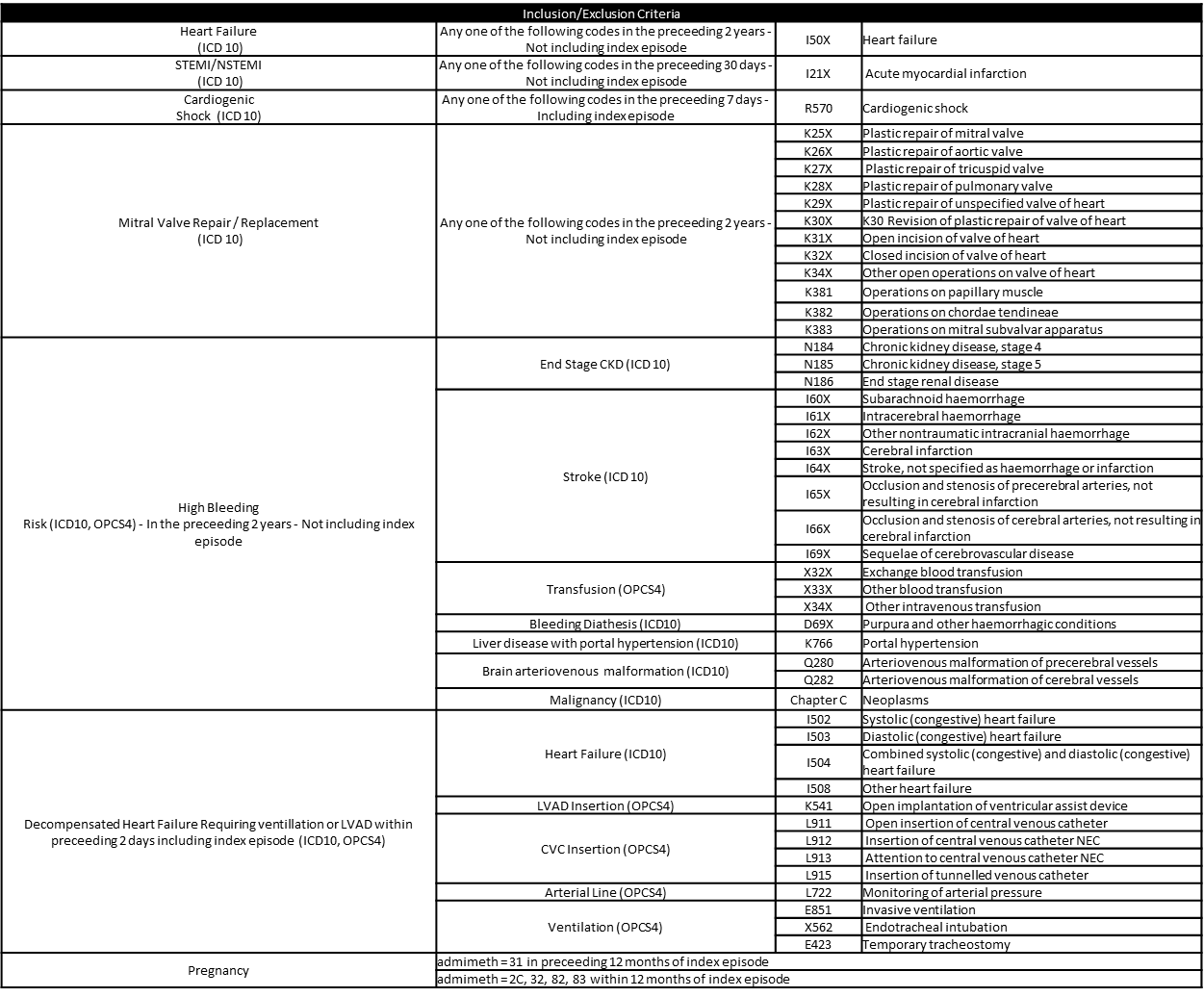


**eTable 2**: ICD-10 and OPCS-4 codes used to phenotype the inclusion and exclusion criteria and define the targeted patient cohort.


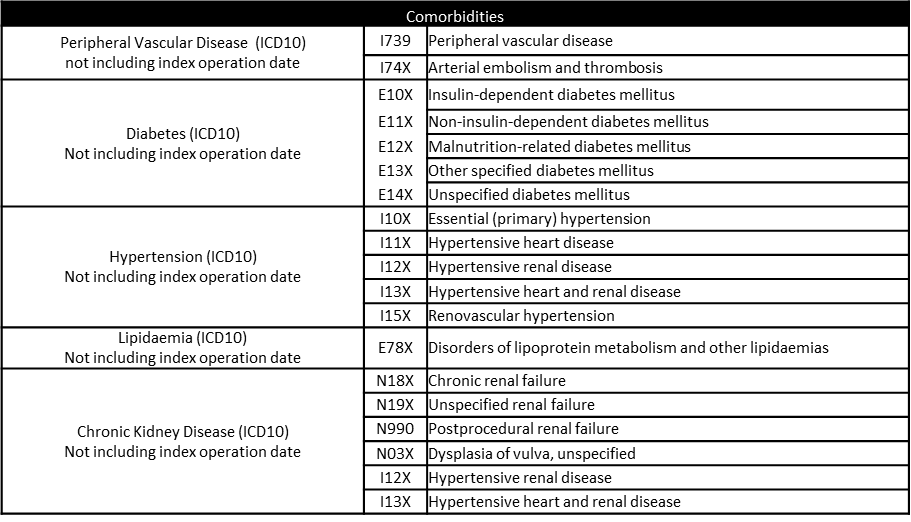


**eTable 3**: ICD-10 used to phenotype patient comorbidities.


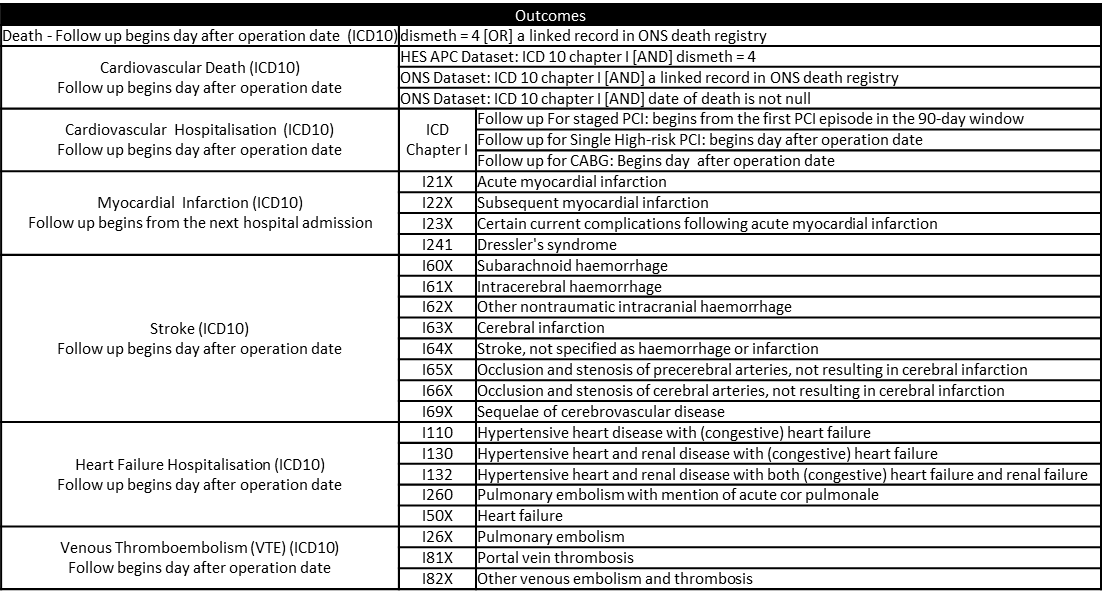


**eTable 4**: For the primary analysis, primary diagnoses were used to phenotype the outcomes. For the sensitivity analysis, both primary and secondary diagnoses were used to phenotype the outcomes.

**2.0 Meta-learner algorithms**

Kunzel et al. first described meta-learning strategies to estimate treatment effects in 2019, these normally consist of two stages[1, 2]:

(1) Base learners are used to build models to estimate conditional expectations of the outcomes for both control and treatment groups.

(2) The difference of these estimates are then averaged across the entire population to estimate the conditional average treatment effect (CATE).

Using ensemble methods such as meta-learners has several additional benefits when compared to traditional methods in causal inference, (1) meta-learners allow for the possibility that the size of the unobservable, within-person, treatment effect may vary as an unknown function of the covariates, and do not assume an underlying distribution (2) meta-learners are generic algorithms refraining from a specific usage of any particular machine learning method. This allows for the application of any suitable supervised learning method for the particular prediction problem at hand. (3) They can be used to estimate heterogeneity of treatment effects and therefore allow better targeting of interventions.[2]

In this analysis extreme gradient boosting (XGBoost) was used as the base learner for three separate meta-learning algorithms (S-Learner, T-Learner and X-Learner) to estimate the effect of treatment allocation on the dichotomous composite outcome of 5-year cardiovascular hospitalisation or mortality. XGBoost is a decision-tree-based ensemble Machine Learning algorithm that uses a gradient boosting framework. Rigorous benchmarking analyses have consistently demonstrated that XGBoost models offer the best combination of prediction performance and processing and was therefore used in this analysis.[3-7]

The S-Learner (eFigure1) includes the treatment indicator as an additional feature in the model. Predictions are then made conditionally under different treatment regimes, which are then used to estimate the treatment effects. The S-learner has the added advantage that it can be implemented with both continuous and discrete treatments, whilst the X and T learner can only be used with discrete treatments. Previous studies have shown that the S- learner tends to bias the treatment effects towards zero, resulting in a smaller treatment effect than the actual true causal effect and is therefore used when the expected average treatment effect is large.[2, 8, 9]

The T-Learner (eFigure2) fits two separate models for the treated and control groups independently. Counterfactual predictions are then made for each treatment in order to estimate the CATE. The T learner performs well when treatment effect is small, heterogenous and the response surfaces of the outcomes under treatment and control are expected to be very different

The X-learner (eFigure3) is a two-stage estimator which is an extension of the T-learner. During stage 1, two separate models are fitted for the treated and control cohorts independently. In the second stage, individual treatment effects are then imputed using both predictive data from the models developed in stage 1, and the actual observed outcomes. Two new models are then subsequently fitted to estimate the individual treatment effects, which are then weighted by a propensity score, to give estimates of CATE. The X-Learner is particularly effective when the average treatment effect is expected to be small or when one treatment group is much larger than the other.[2]

Treatment effects for all meta learners were expressed as a risk difference with 95% confidence intervals (average treatment effect).


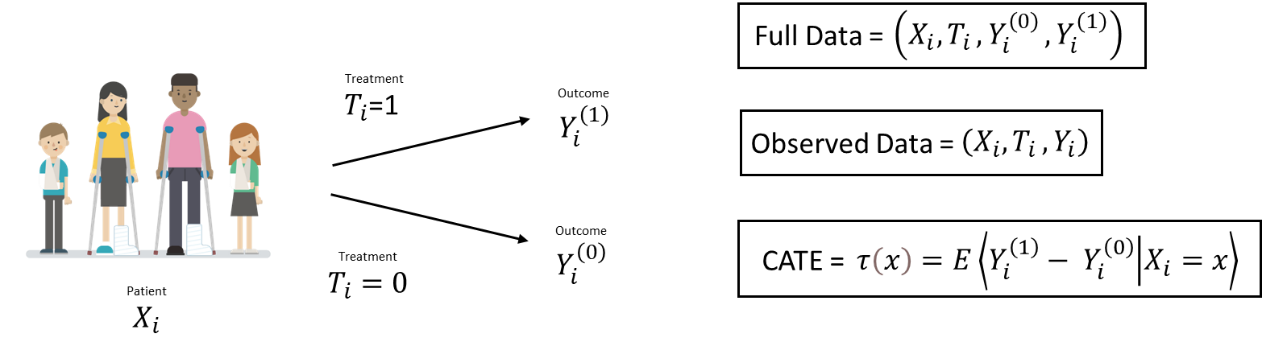

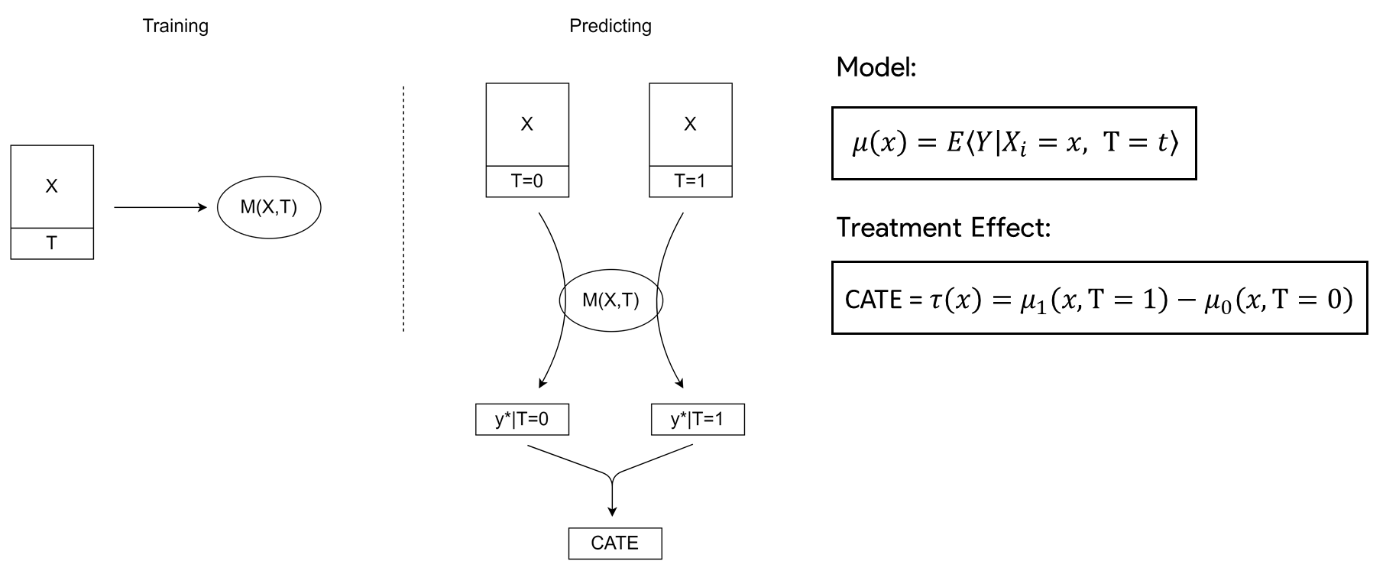


**eFigure 1**: Parameter Definitions

**eFigure 2**: S-learner Algorithm for conditional average treatment effect prediction


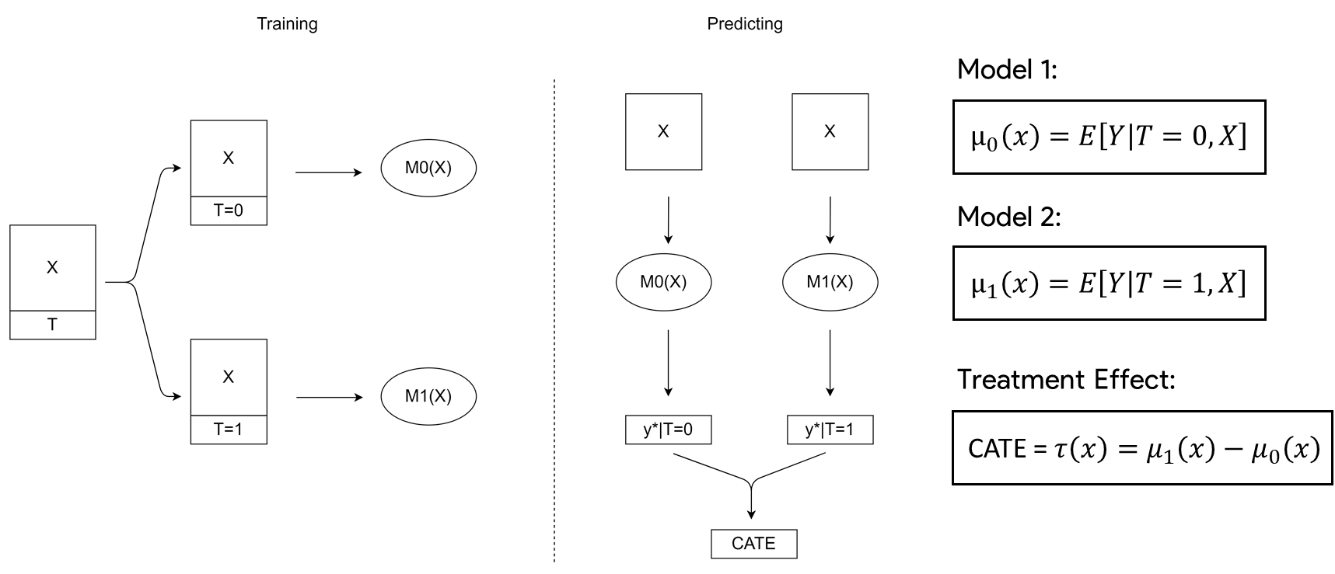


**eFigure 3**: T-learner Algorithm for conditional average treatment effect prediction


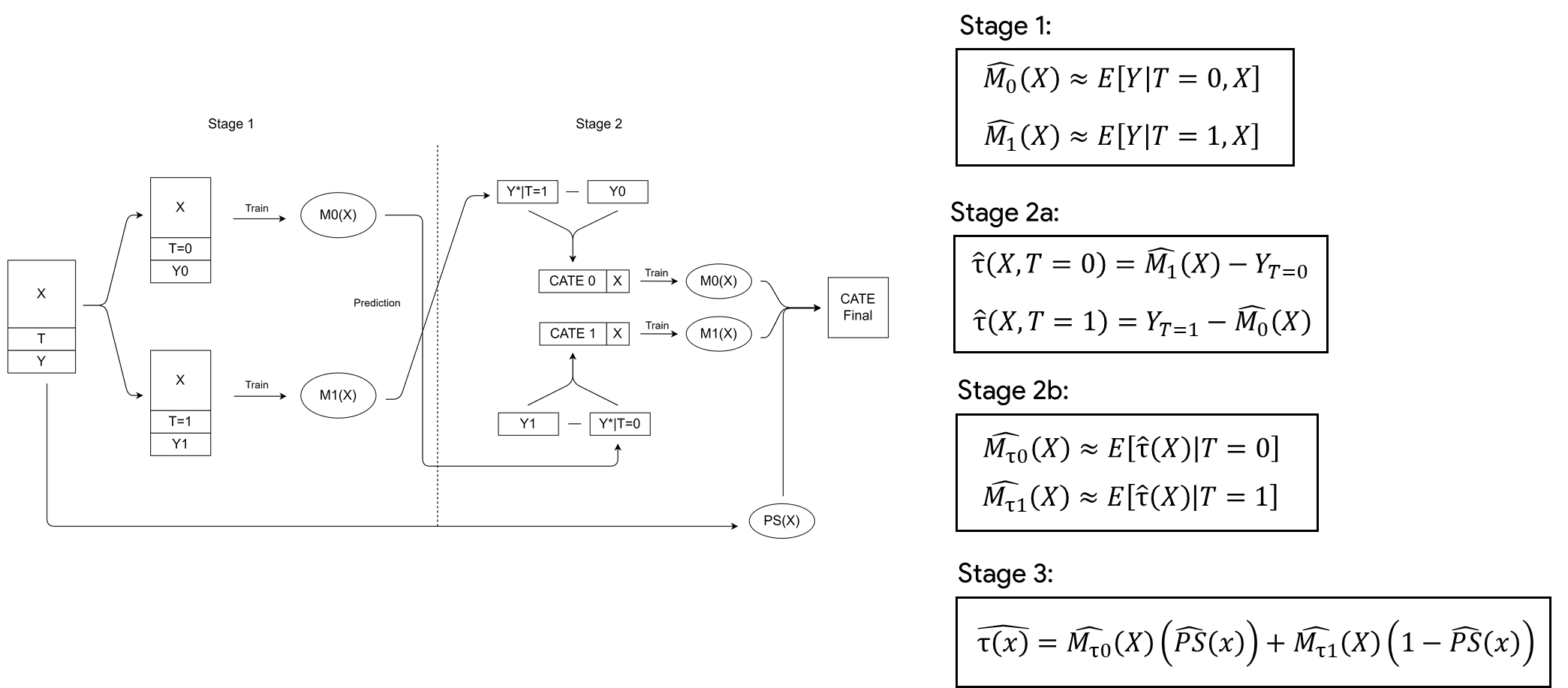


**eFigure 4**: X-learner Algorithm for conditional average treatment effect prediction


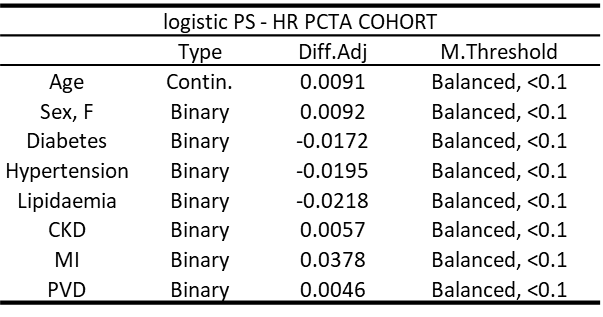


**eTable 5**: Matching algorithm performance: Logistic Propensity Score Matching - PTCA cohort


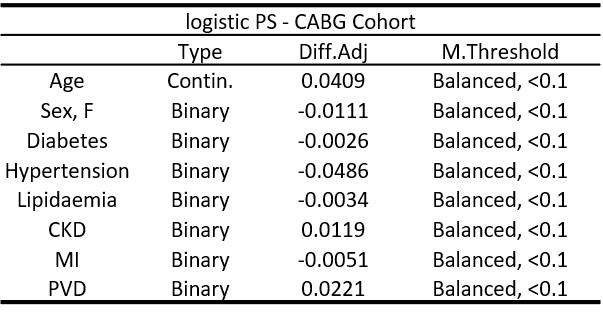


**eTable 6**: Matching algorithm performance: Logistic propensity score matching - CABG cohort


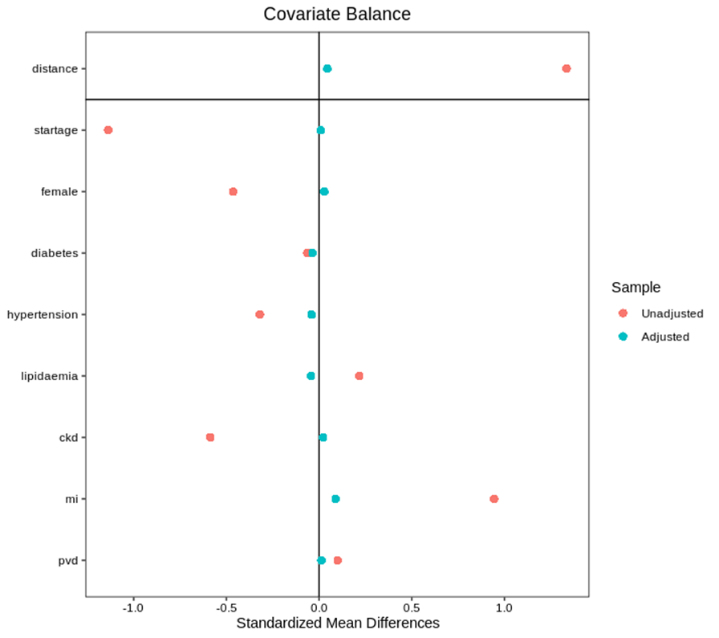


**eFigure5**: Balance Plot: Logistic Propensity Score Matching - PTCA cohort


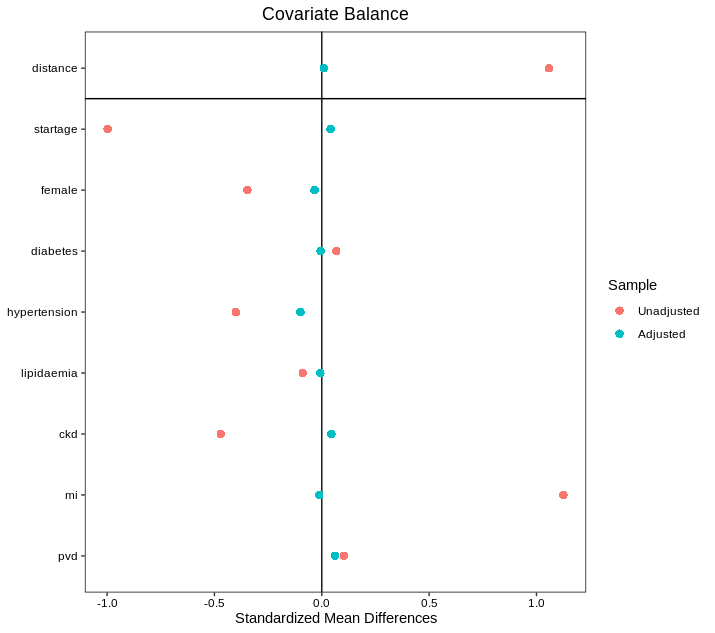


**eFigure6**: Balance Plot: Logistic Propensity Score matching - CABG cohort

**3.0 Matching Algorithms**

**4.0 Patient Characteristics by Quartile of the Regional Surgical Rate**

|  |  | **IVA Quartile 1** | **IVA Quartile 2** | **IVA Quartile 3** | **IVA Quartile 4** |
| --- | --- | --- | --- | --- | --- |
| **n** |  | 3398 | 3365 | 3272 | 3456 |
| **Regional Surgical Rates, median (IQR)** |  | 65.0 (58.0, 70.0) | 77.0 (76.0, 79.0) | 83.0 (81.0, 84.0) | 90.0 (88.0, 91.0) |
| **Age, median (IQR)** |  | 71.0 (63.0, 78.0) | 71.0 (63.0, 77.0) | 71.0 (64.0, 77.0) | 72.0 (64.0, 77.0) |
| **Ethnicity** | **White** | 2699 (79.4%) | 2644 (78.6%) | 2792 (85.3%) | 3096 (89.6%) |
|  | **Asian** | 319 (9.4%) | 327 (9.7%) | 171 (5.2%) | 75 (2.2%) |
|  | **Black** | 55 (1.6%) | 36 (1.1%) | 12 (0.4%) | 9 (0.3%) |
|  | **Mixed / Other** | 76 (2.2%) | 73 (2.2%) | 31 (0.9%) | 29 (0.8%) |
|  | **NA** | 249 (7.3%) | 285 (8.5%) | 266 (8.1%) | 247 (7.1%) |
| **sex** | **Male** | 2527 (74.4%) | 2553 (75.9%) | 2532 (77.4%) | 2591 (75.0%) |
|  | **Female** | 871 (25.6%) | 812 (24.1%) | 740 (22.6%) | 865 (25.0%) |
| **Index of Multiple Deprivation, median (IQR)** |  | 18.8 (10.6, 32.9) | 18.9 (10.4, 32.0) | 18.8 (11.1, 30.4) | 16.4 (10.0, 26.9) |
| **Diabetes** |  | 1380 (40.6%) | 1346 (40.0%) | 1198 (36.6%) | 1135 (32.8%) |
| **Hypertension** |  | 2718 (80.0%) | 2735 (81.3%) | 2574 (78.7%) | 2612 (75.6%) |
| **Lipidaemia** |  | 2017 (59.4%) | 2191 (65.1%) | 2038 (62.3%) | 2051 (59.3%) |
| **Chronic Kidney Disease** |  | 767 (22.6%) | 717 (21.3%) | 687 (21.0%) | 666 (19.3%) |
| **Stroke** |  | 56 (1.6%) | 85 (2.5%) | 79 (2.4%) | 69 (2.0%) |
| **Myocardial Infarction** |  | 1014 (29.8%) | 1094 (32.5%) | 1040 (31.8%) | 1072 (31.0%) |
| **Peripheral Vascular Disease** |  | 422 (12.4%) | 387 (11.5%) | 362 (11.1%) | 384 (11.1%) |
| **HFRS, median (IQR)** |  | 11.5 (5.1, 21.6) | 11.3 (4.8, 21.6) | 9.6 (4.1, 18.8) | 9.5 (4.2, 18.8) |
| **CCI, median (IQR)** |  | 7.0 (4.0, 12.0) | 7.0 (4.0, 11.0) | 6.0 (4.0, 10.0) | 6.0 (3.0, 10.0) |

**CCI:** Charlson Comorbidity Index

**IMD:** Index of Multiple Deprivation

**HFRS:** Hospital Frailty Risk Score

**eTable 7:** Baseline characteristics across quartiles of regional surgical rates.


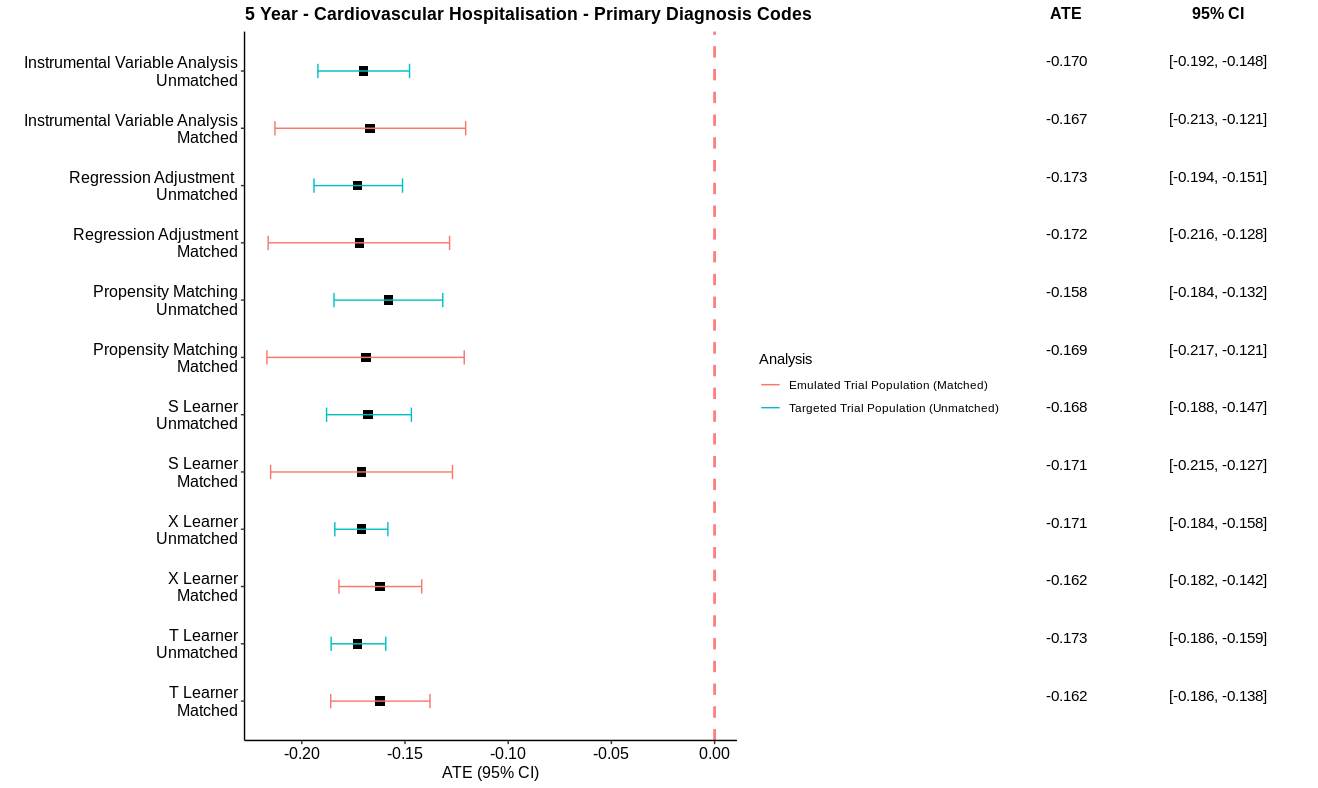


**5.0 Secondary Outcomes - Estimated Treatment Effects - Primary Diagnosis Codes**

**eFigure 7**: Average Treatment effect with 95% CI for **cardiovascular hospitalisation** at 5 years follow up for both matched and unmatched cohorts. Only Primary diagnosis (PD) codes used. Treatment effect left of the reference line favors CABG.


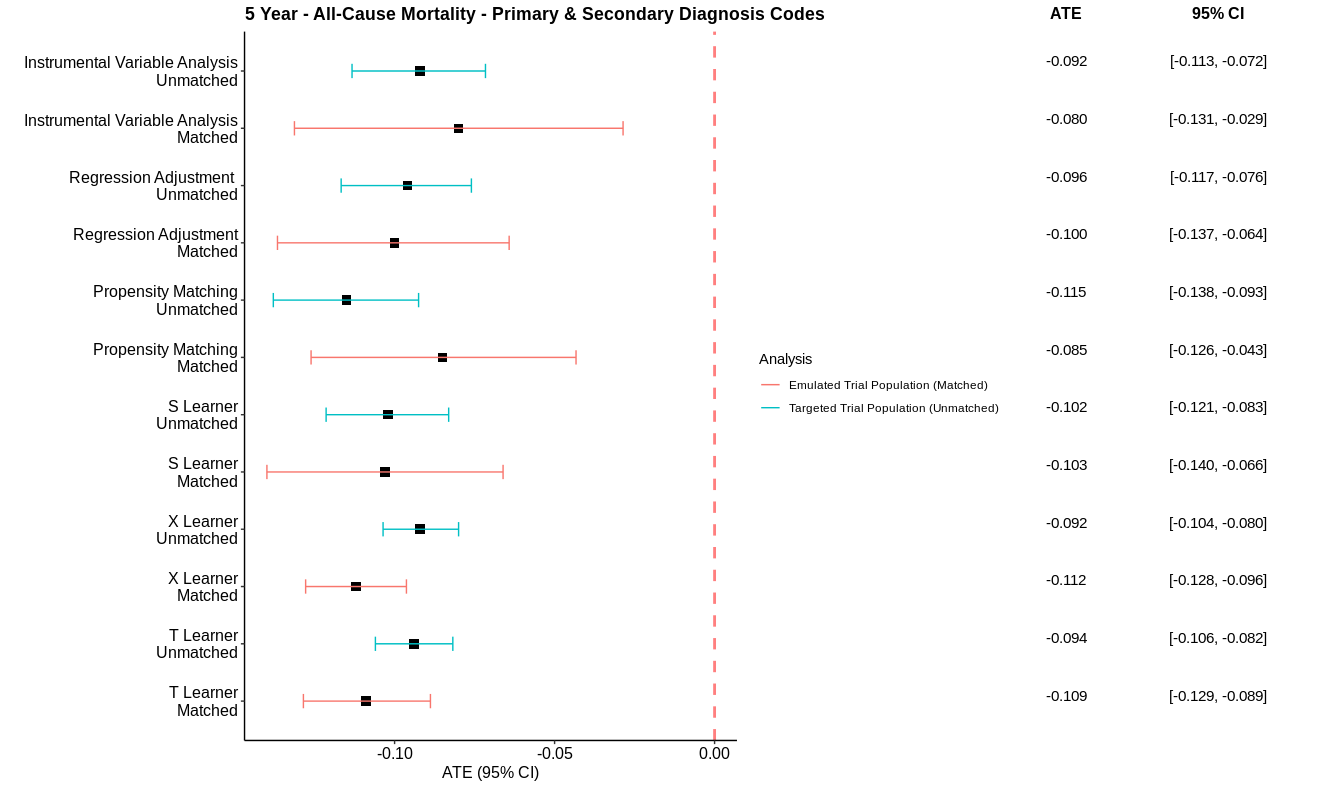

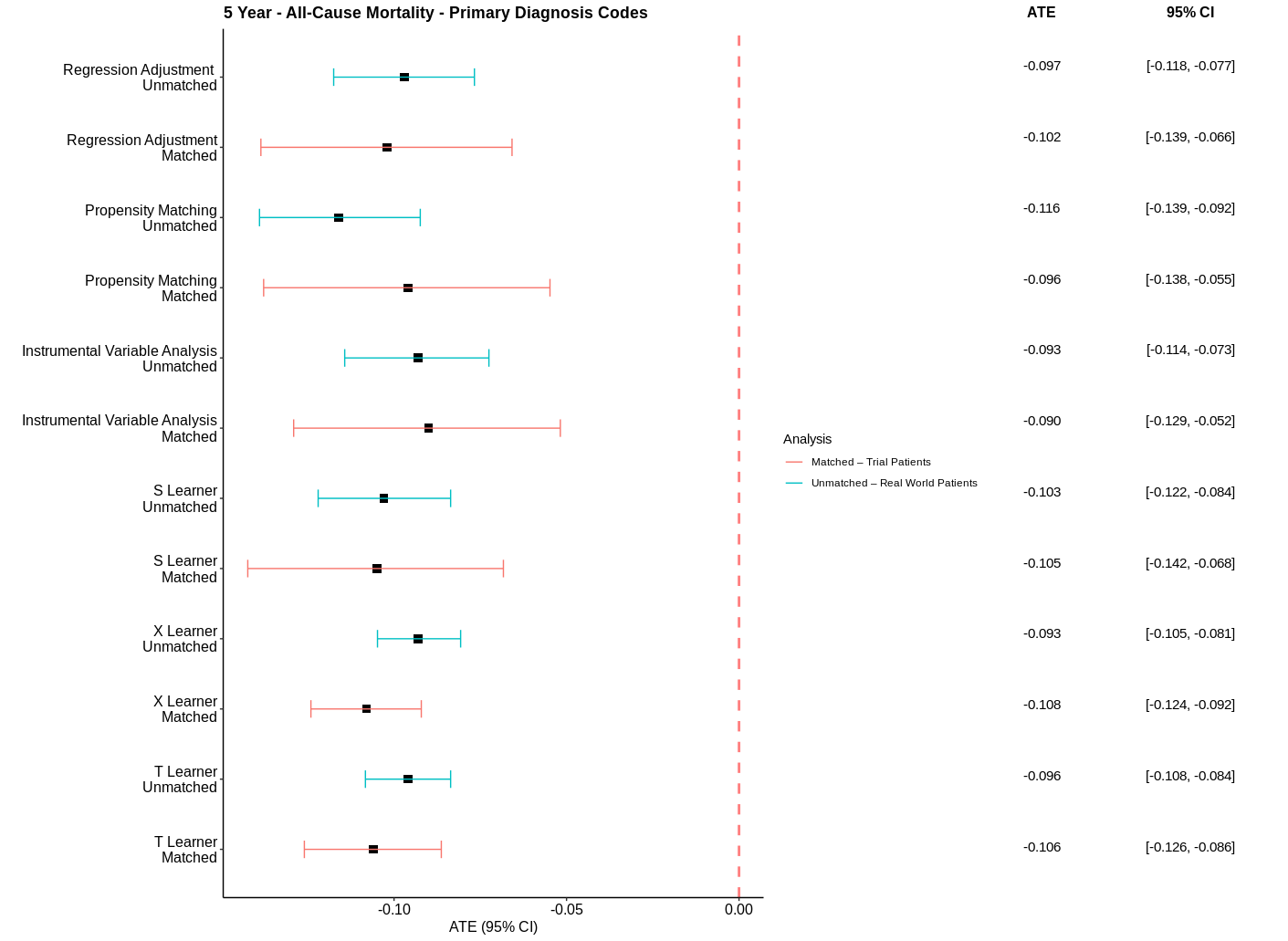


**eFigure 8**: Average Treatment effect with 95% CI for **all-cause mortality** at 5 years follow up for both matched and unmatched cohorts. Treatment effect left of the reference line favors CABG.


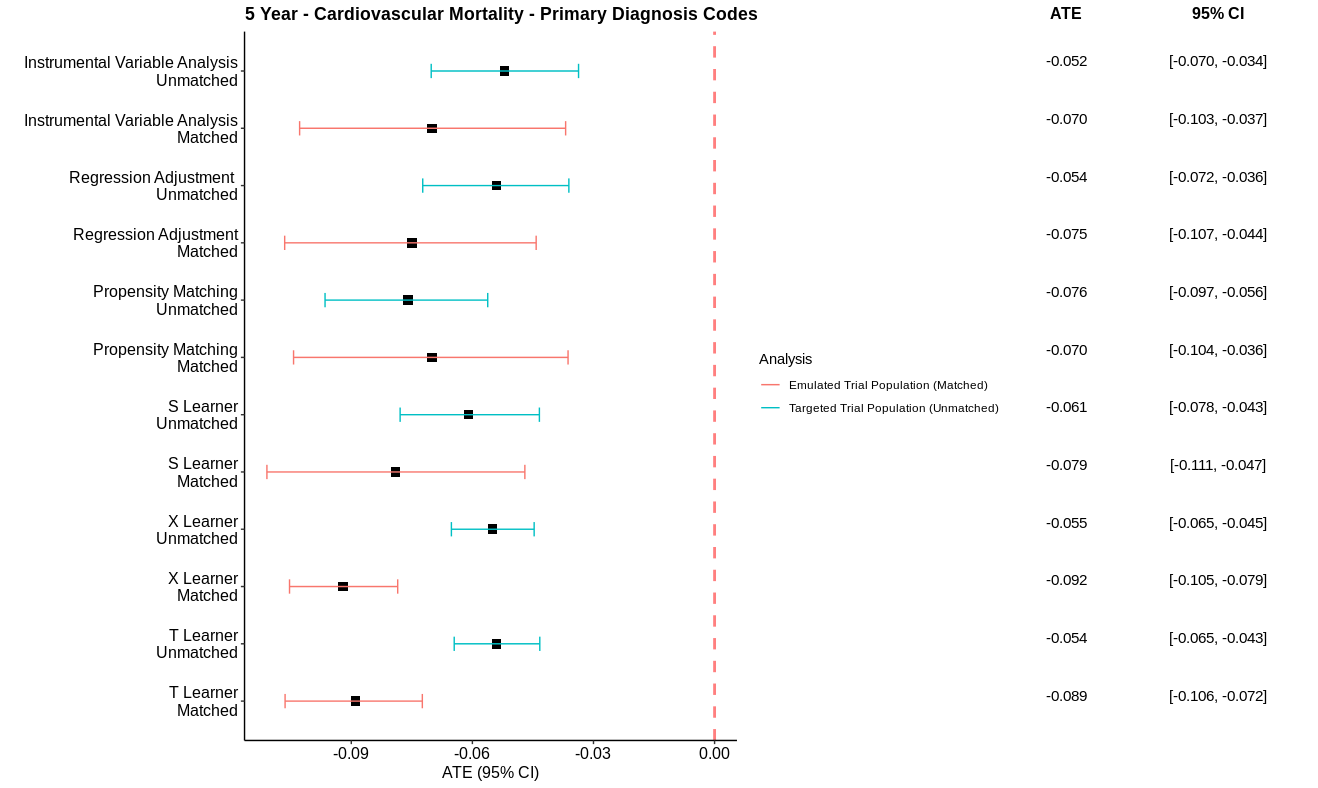


**eFigure 9**: Average Treatment effect with 95% CI for **cardiovascular mortality** at 5 years follow up for both matched and unmatched cohorts. Only Primary diagnosis (PD) codes used. Treatment effect left of the reference line favors CABG.


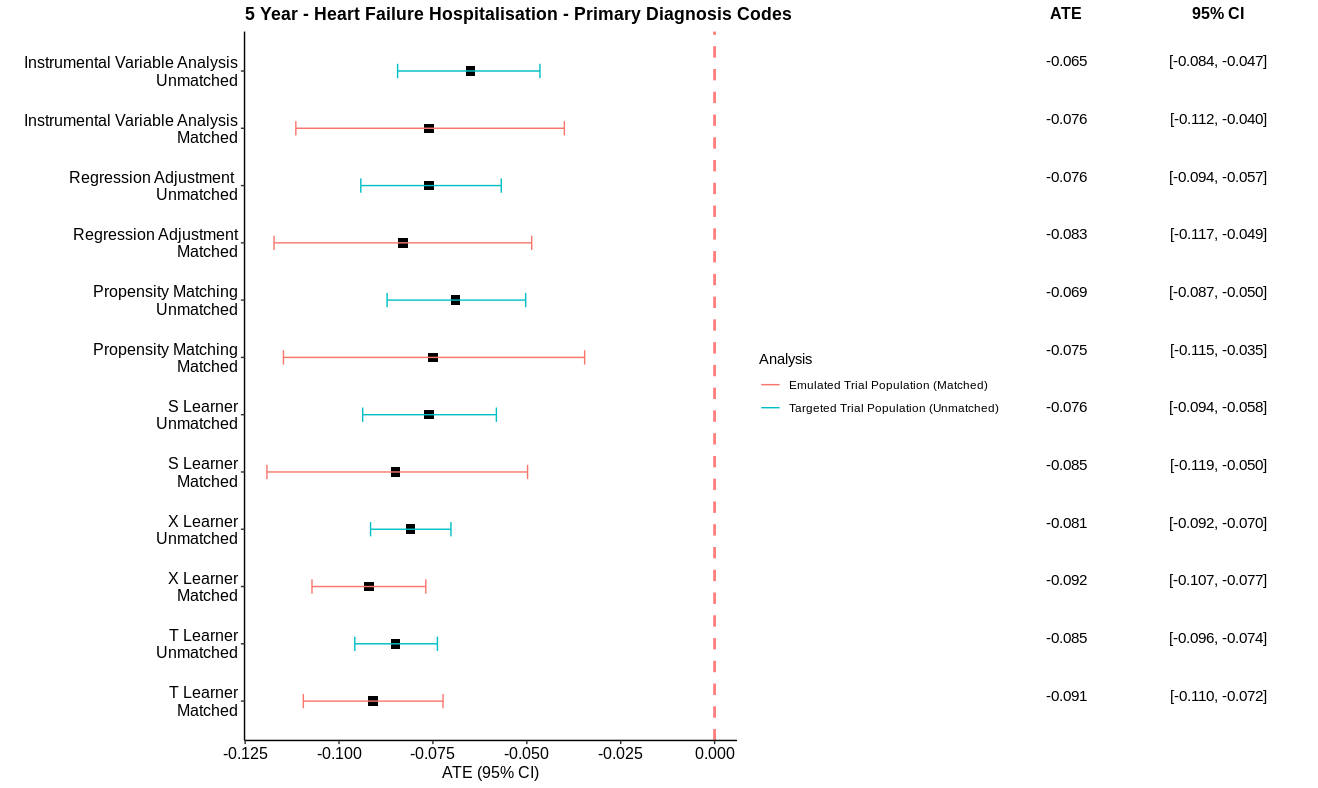


**eFigure 10**: Average Treatment effect with 95% CI for **heart failure hospitalisation** at 5 years follow up for both matched and unmatched cohorts. Only Primary diagnosis (PD) codes used. Treatment effect left of the reference line favors CABG.


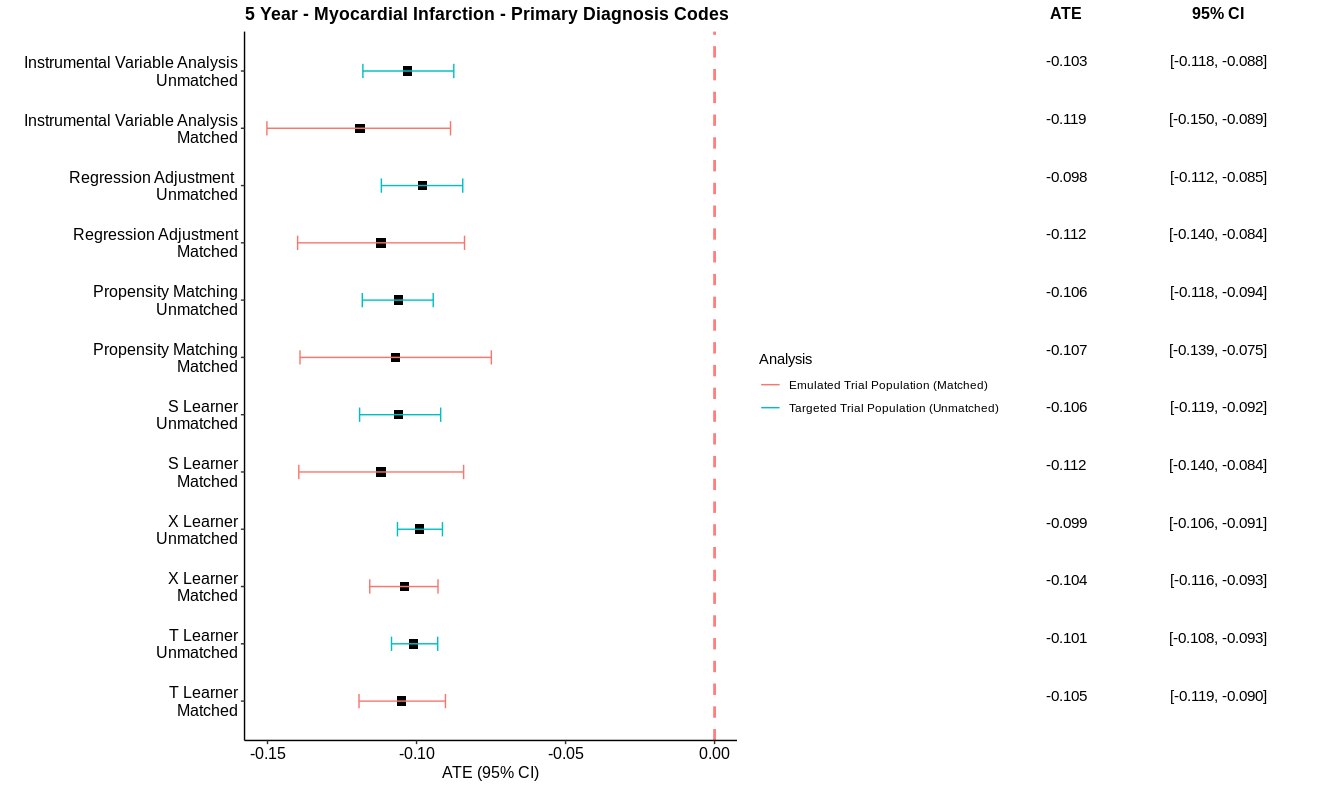


**eFigure 11**: Average Treatment effect with 95% CI for **myocardial infarction** at 5 years follow up for both matched and unmatched cohorts. Only Primary diagnosis (PD) codes used. Treatment effect left of the reference line favors CABG.


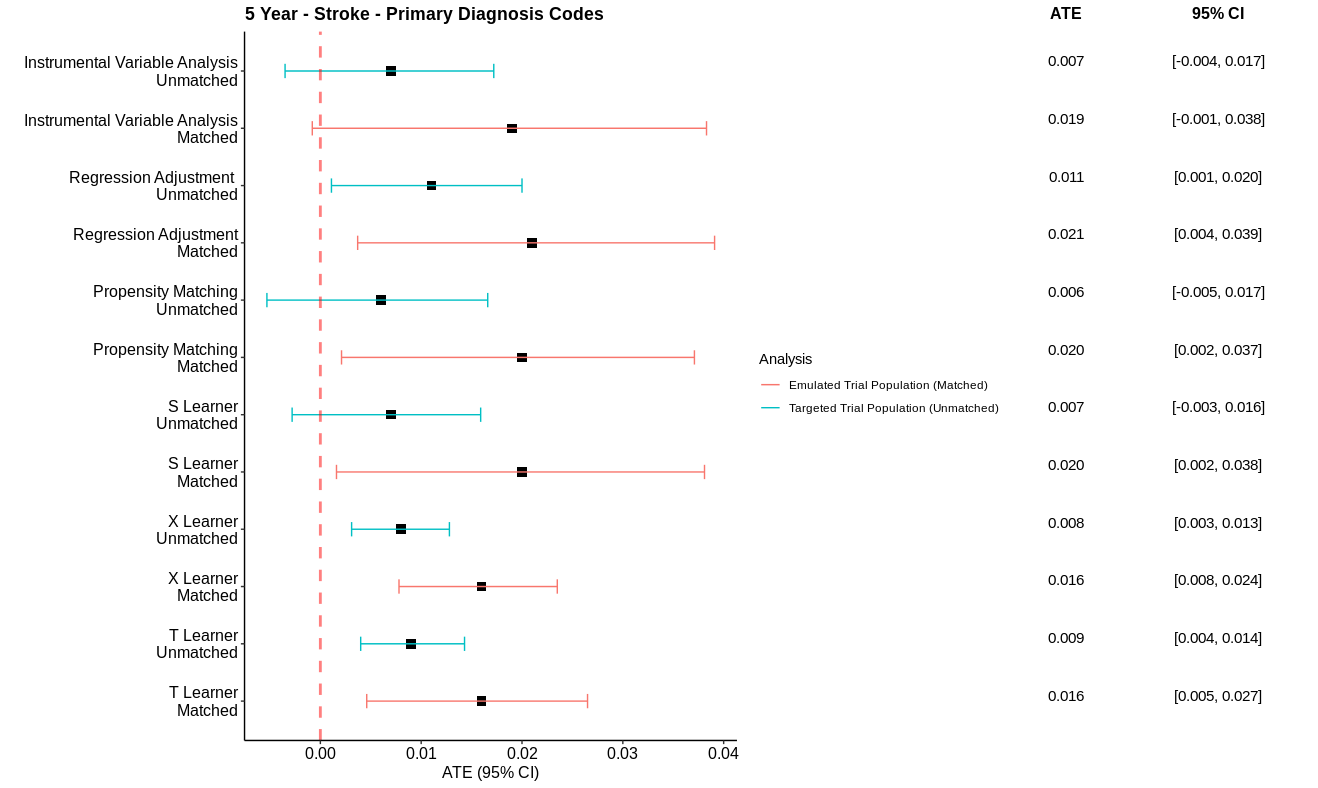


**eFigure 12**: Average Treatment effect with 95% CI for **stroke** at 5 years follow up for both matched and unmatched cohorts. Only Primary diagnosis (PD) codes used. Treatment effect left of the reference line favors CABG.


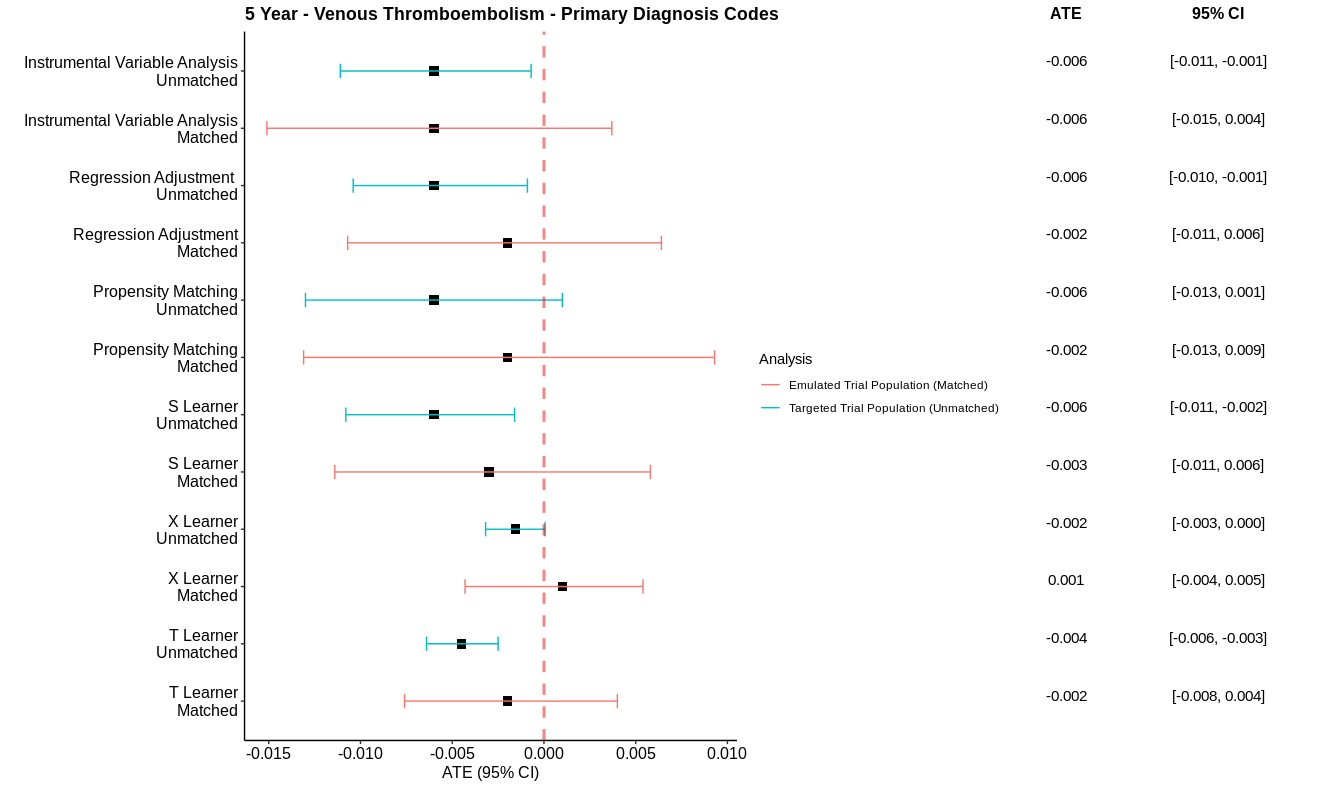


**eFigure 13**: Average Treatment effect with 95% CI for **venous thromboembolism** at 5 years follow up for both matched and unmatched cohorts. Only Primary diagnosis (PD) codes used. Treatment effect left of the reference line favors CABG.


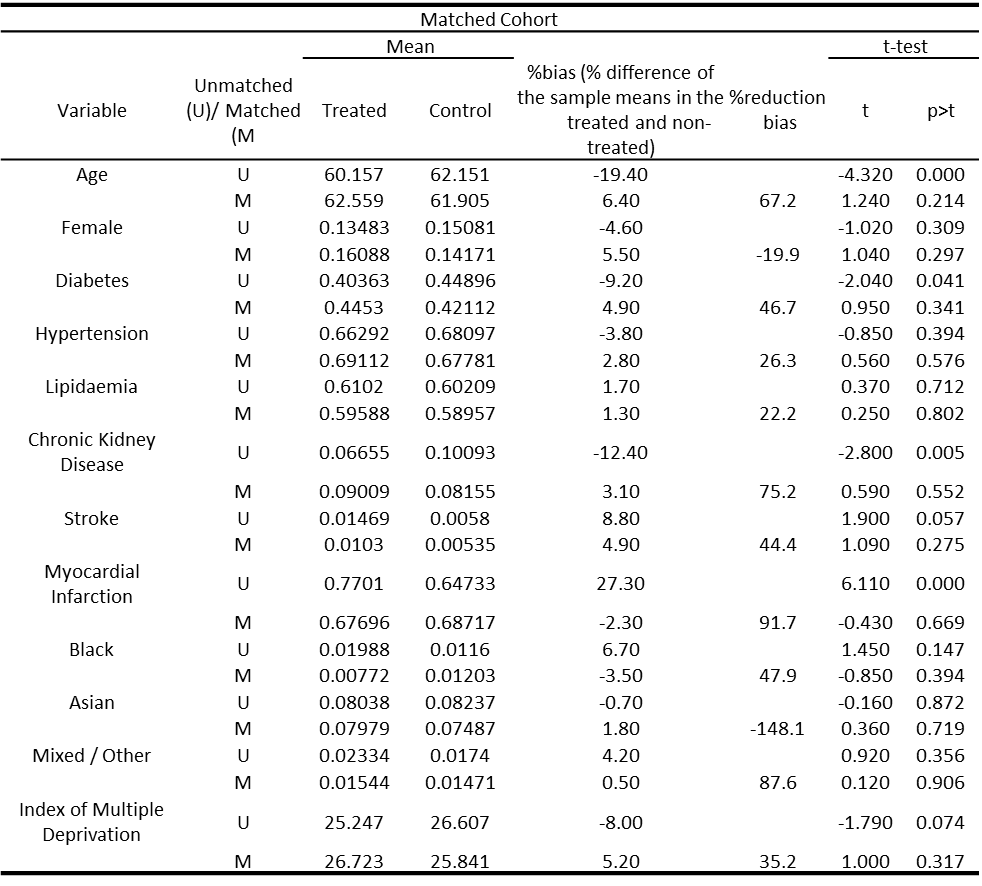

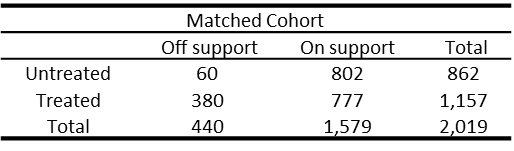


**eFigure 14**: Balance Plot of % difference of covariate means in the treated and non-treated groups, in the matched cohort, using only the primary diagnosis codes, before and after propensity score matching.

**6.0 Propensity Score Matching**

**eTable 8**: Summary of the % difference of covariate means in the treated and non-treated groups, in the matched cohort, using only the primary diagnosis codes, before and after propensity score matching.

**eTable 9**: number of patients in the matched cohort who had overlapping propensity scores (On Support) which were subsequently included in the analysis.

**eFigure 15**: Balance Plot of % difference of covariate means in the treated and non-treated groups, in the unmatched cohort, using only the primary diagnosis codes, before and after propensity score matching.


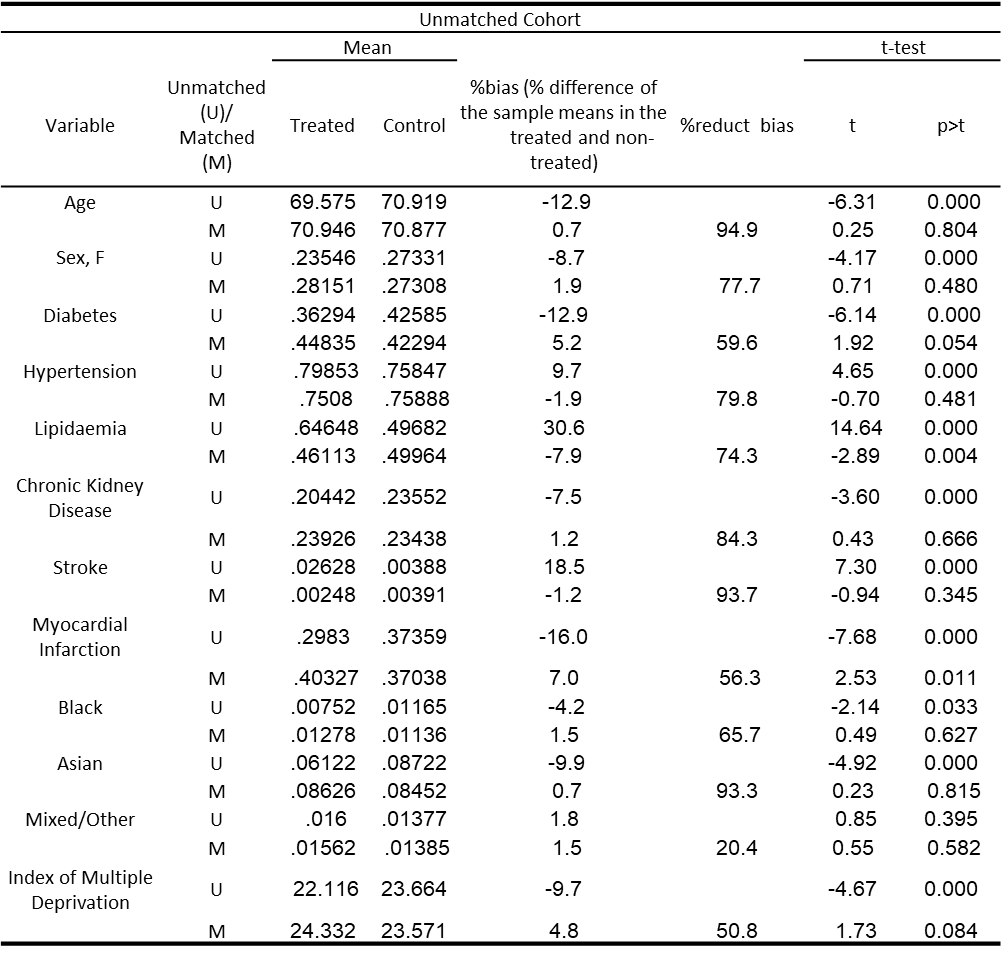


**eTable 10**: Summary of the % difference of covariate means in the treated and non-treated groups, in the unmatched cohort, using only the primary diagnosis codes, before and after propensity score matching.


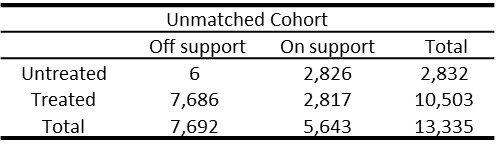


**eTable 11**: number of patients in the unmatched cohort who had overlapping propensity scores (On Support) which were subsequently included in the analysis.


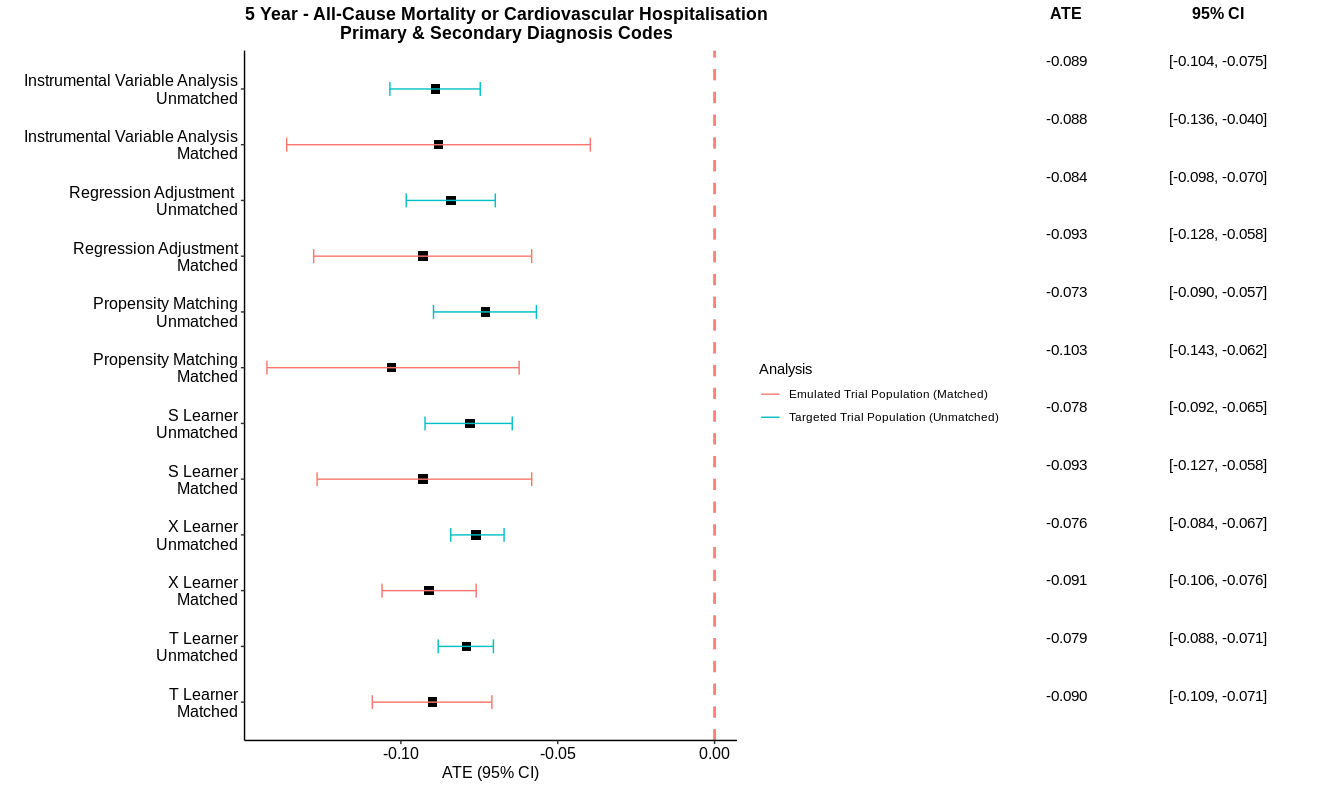


**7.0 Sensitivity Analysis - Estimated Treatment Effects - Primary and Secondary Diagnosis Codes**

**eFigure 15**: Average Treatment effect with 95% CI for **venous thromboembolism** at 5 years follow up for both matched and unmatched cohorts. Only Primary diagnosis (PD) codes used. Treatment effect left of the reference line favors CABG.

**Figure 16**: Average Treatment effect with 95% CI for the primary composite outcome of **mortality or cardiovascular hospitalisation** at 5 years of follow-up for both matched and unmatched cohorts. Both Primary and secondary diagnosis codes were used.


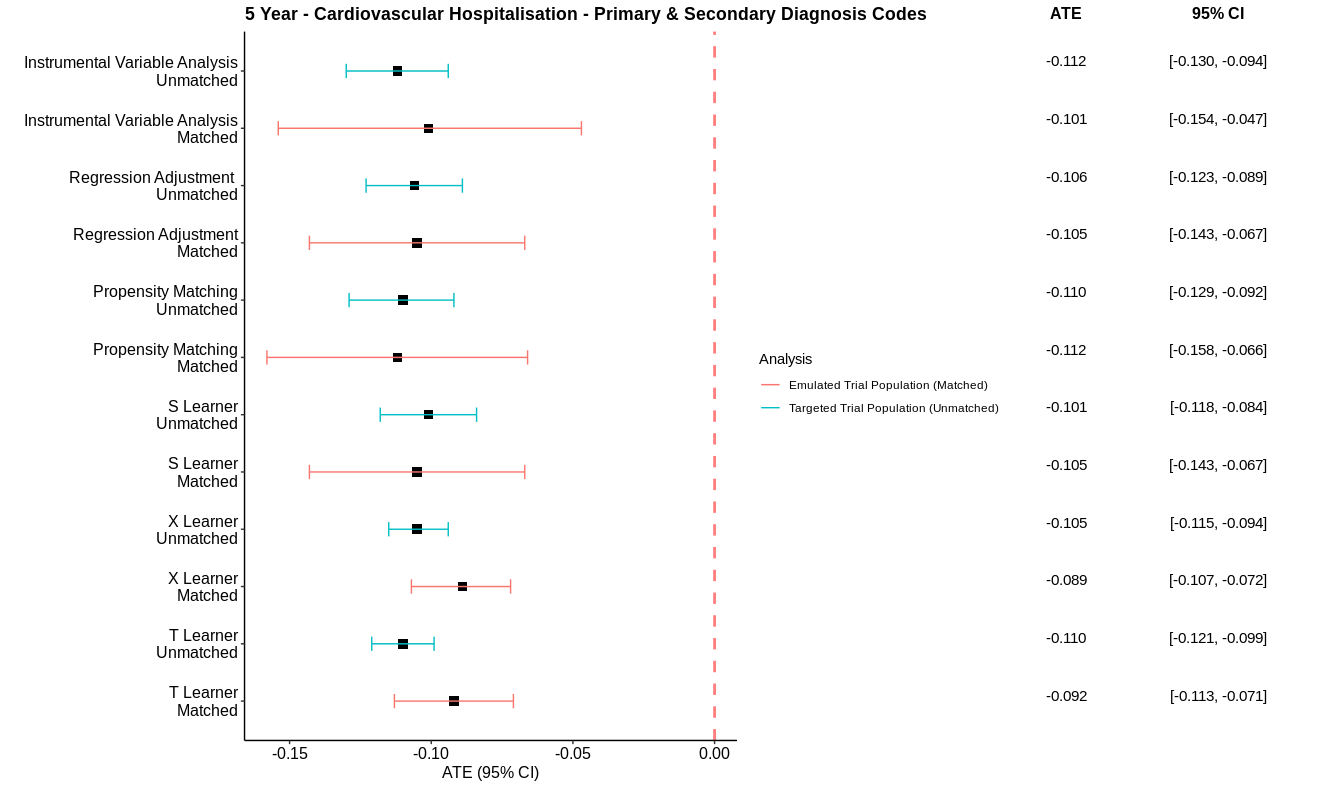


**eFigure 17**: Average Treatment effect with 95% CI for **cardiovascular hospitalisation** at 5 years follow up for both matched and unmatched cohorts. Both primary and secondary diagnosis codes used. Treatment effect left of the reference line favors CABG.


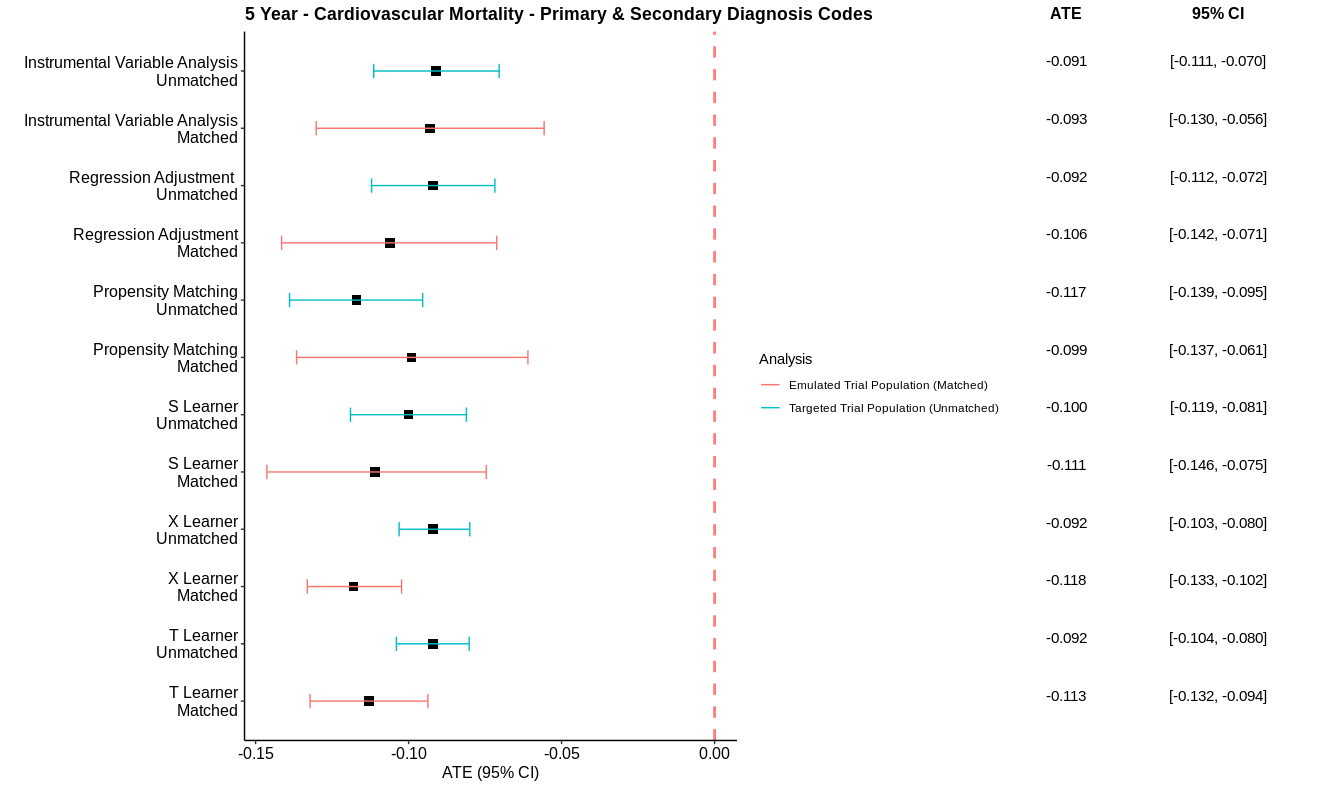


**eFigure 18**: Average Treatment effect with 95% CI for **cardiovascular mortality** at 5 years follow up for both matched and unmatched cohorts. Both primary and secondary diagnosis codes used. Treatment effect left of the reference line favors CABG.


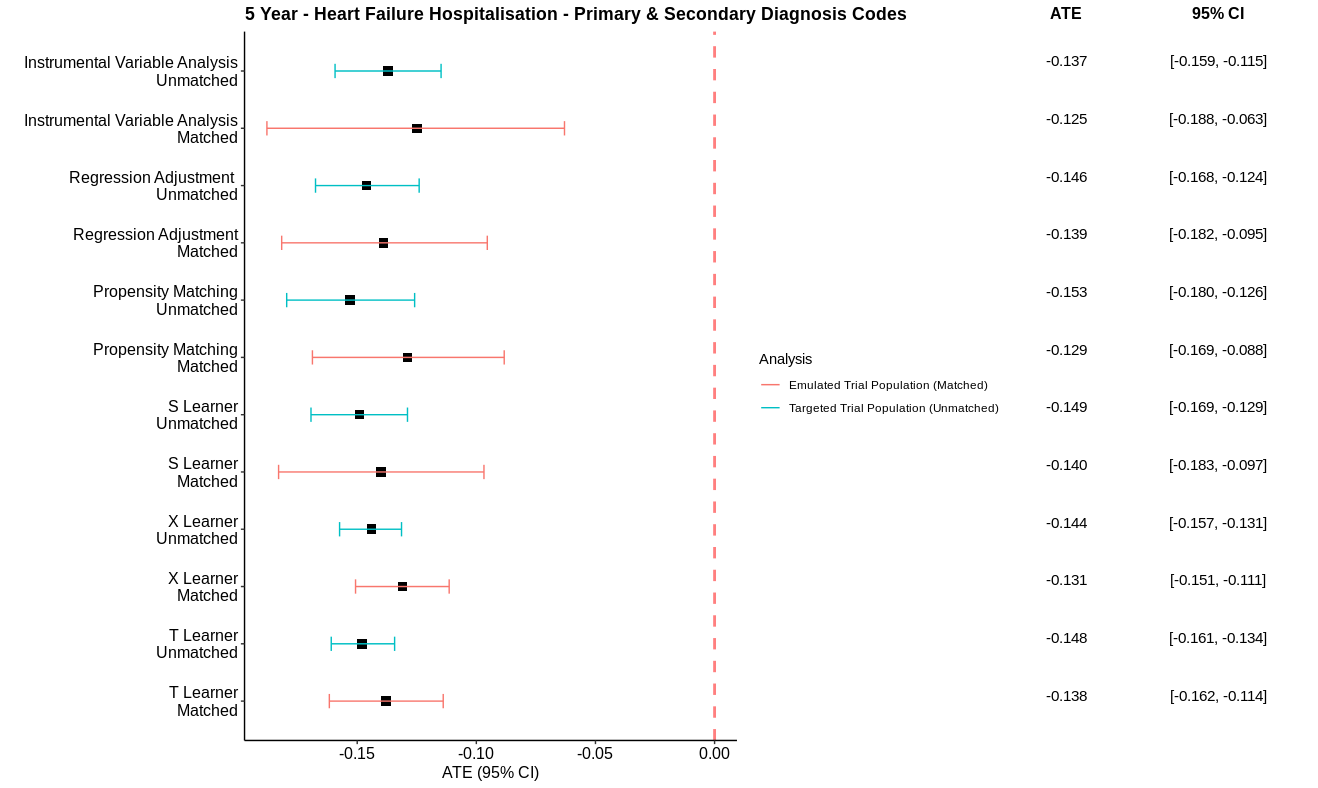


**eFigure 19**: Average Treatment effect with 95% CI for **heart failure hospitalisation** at 5 years follow up for both matched and unmatched cohorts. Both primary and secondary diagnosis codes used. Treatment effect left of the reference line favors CABG.


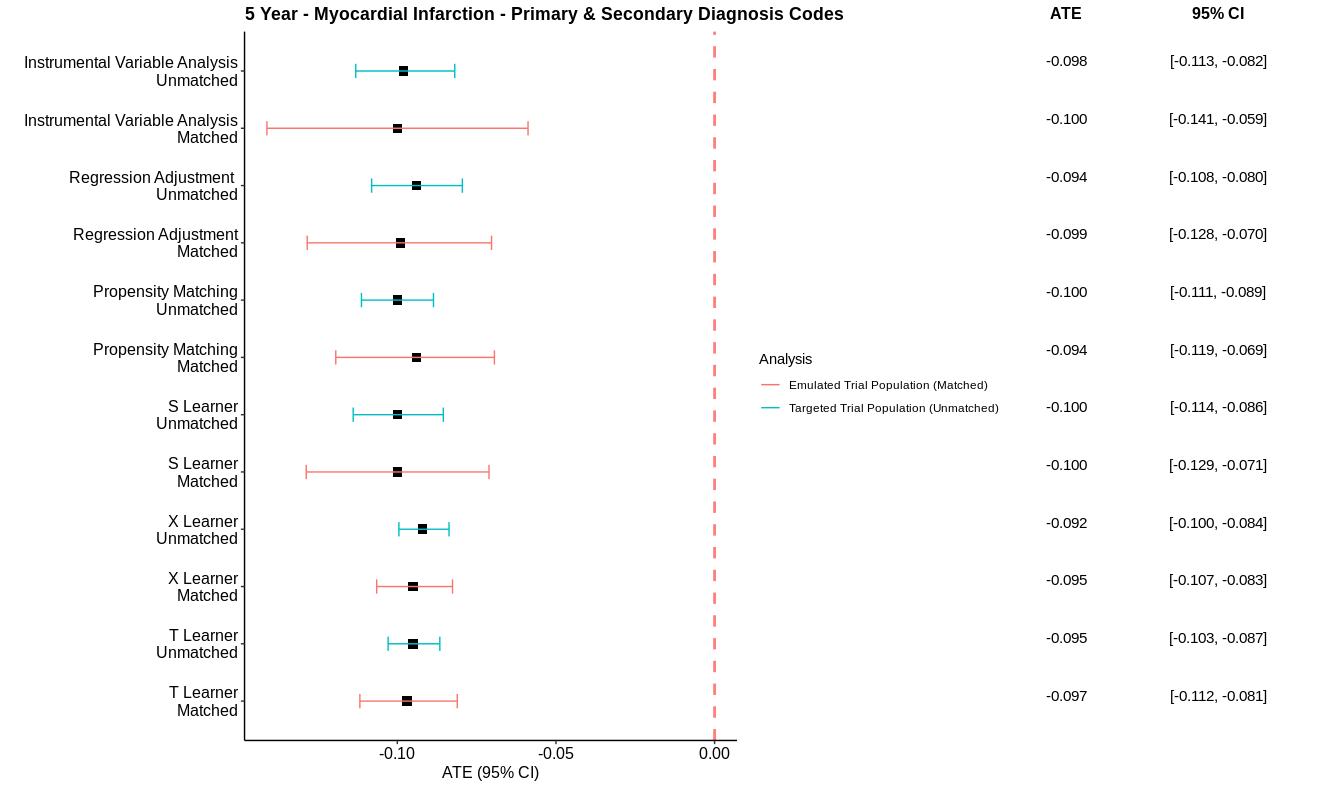


**eFigure 20**: Average Treatment effect with 95% CI for **myocardial infarction** at 5 years follow up for both matched and unmatched cohorts. Both primary and secondary diagnosis codes used. Treatment effect left of the reference line favors CABG.


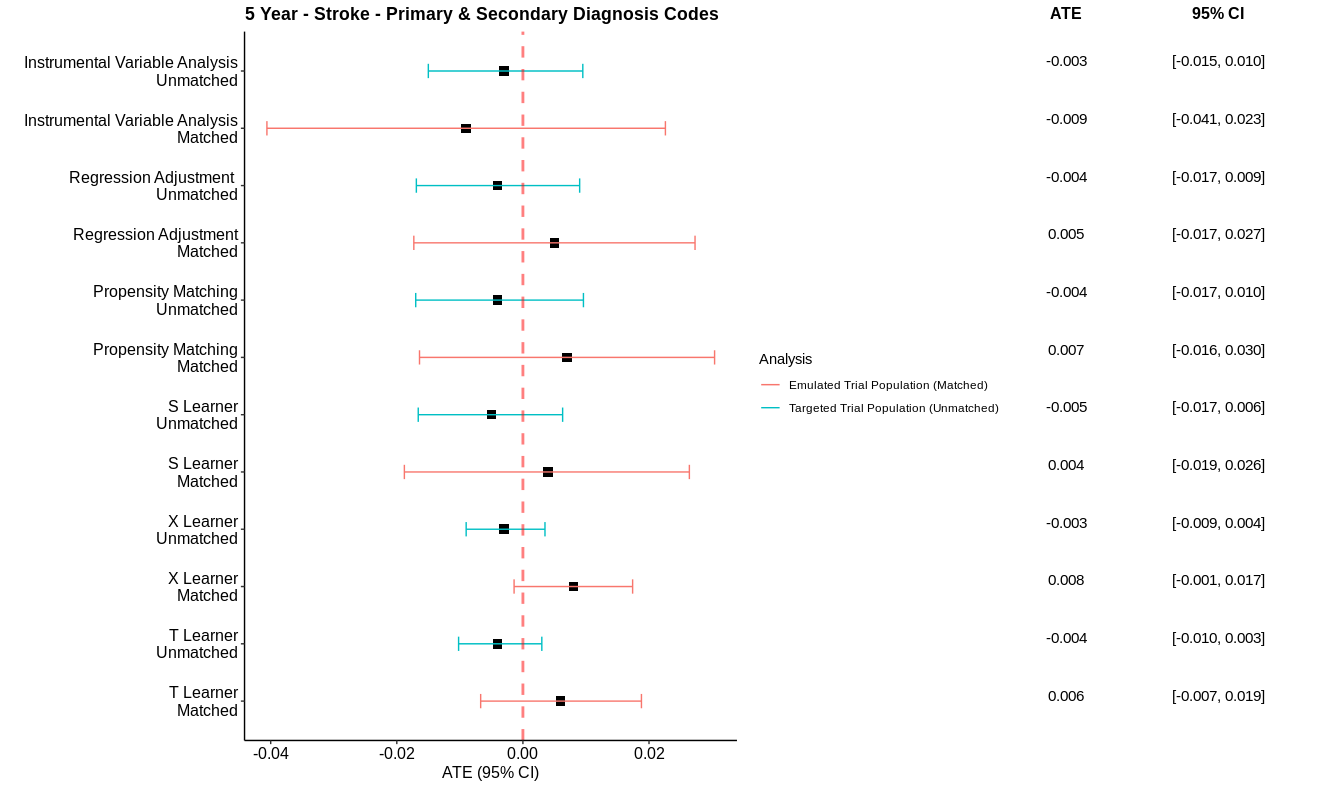


**eFigure 21**: Average Treatment effect with 95% CI for **stroke** at 5 years follow up for both matched and unmatched cohorts. Both primary and secondary diagnosis codes used. Treatment effect left of the reference line favors CABG.


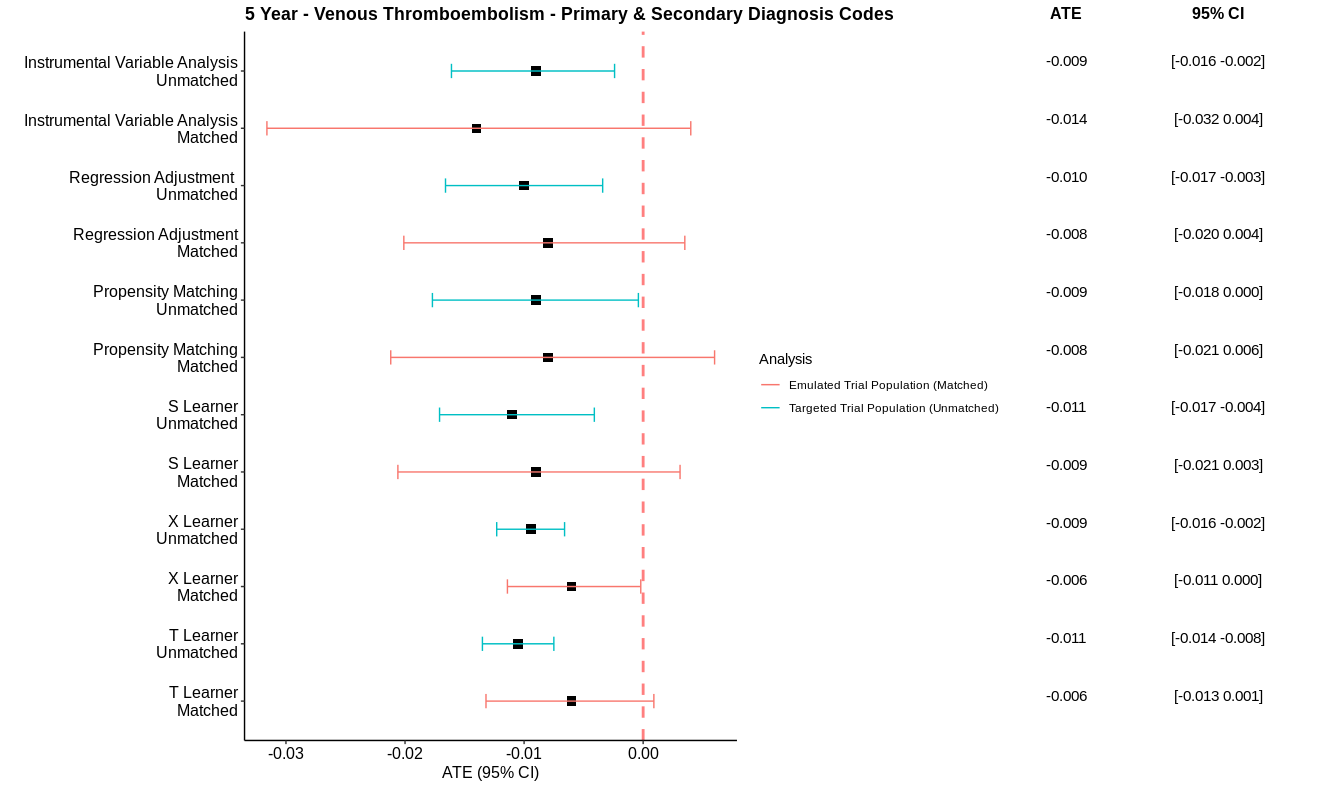


**eFigure 22**: Average Treatment effect with 95% CI for **venous thromboembolism** at 5 years follow up for both matched and unmatched cohorts. Both primary and secondary diagnosis codes used. Treatment effect left of the reference line favors CABG.


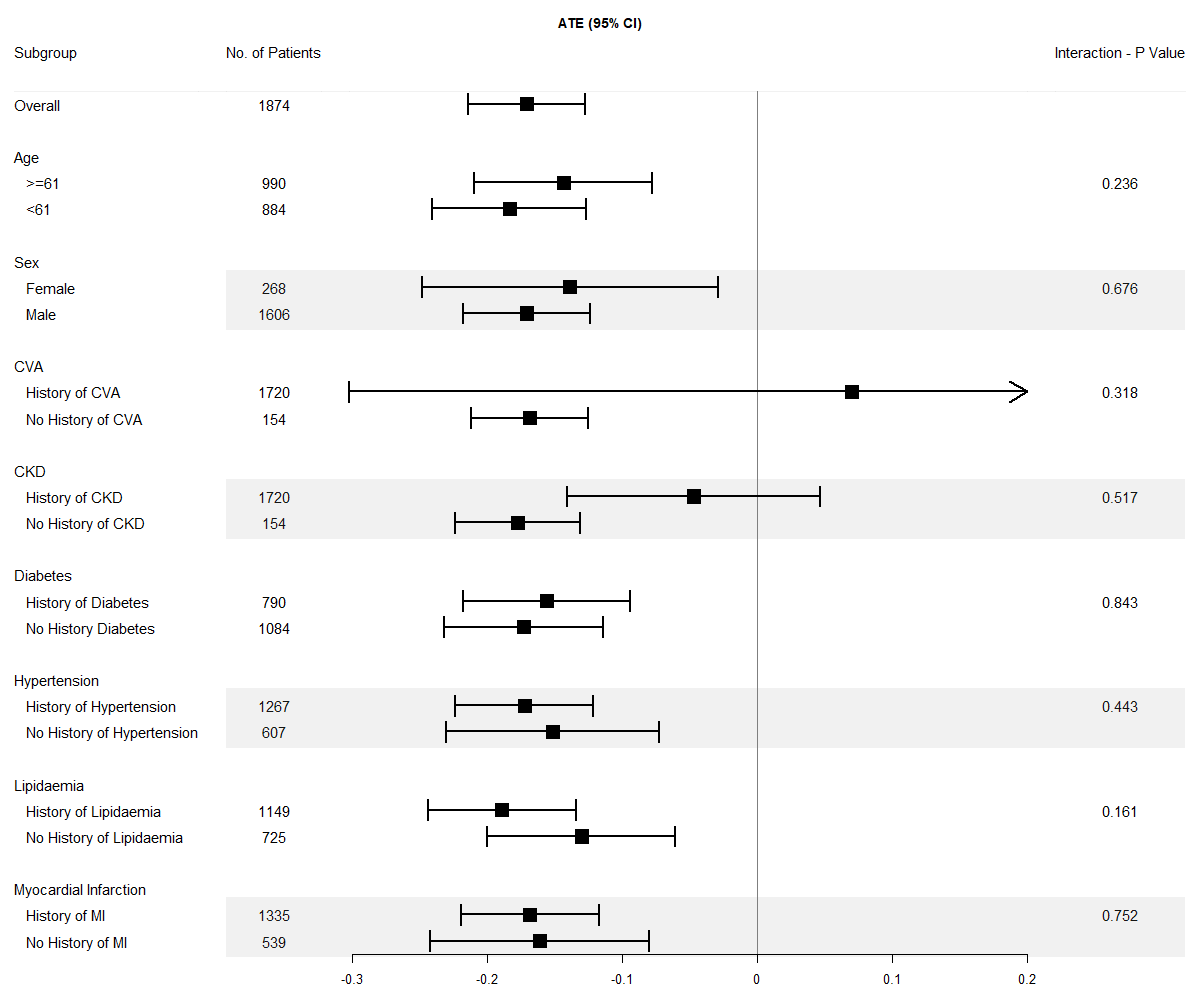


**8.0 Subgroup Analysis – Primary Outcome**

**eFigure 23**: Average Treatment effect with 95% CI for our cohort stratified by clinically important comorbidities. Treatment effect left of the reference line favors CABG.

**9.0 Sample Size Sensitivity Analysis**


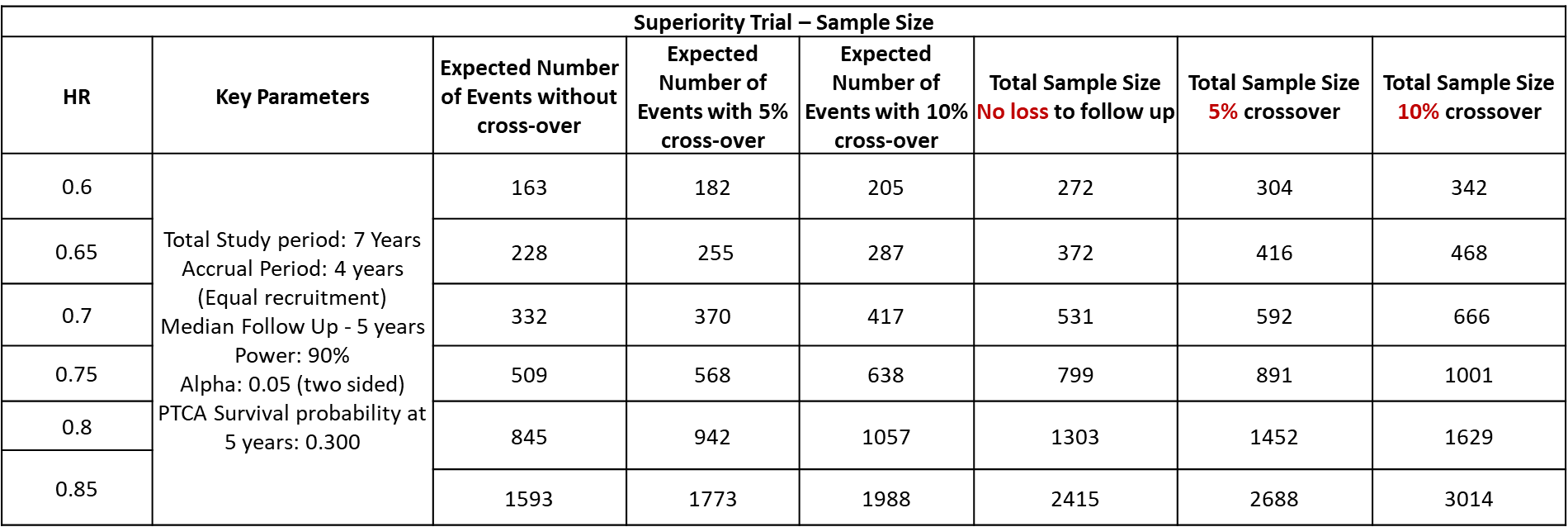


**eTable12**: Sample size estimates for a range of minimum clinically important treatment effects.

**References**

1. Curth, A. and M. van der Schaar. *Nonparametric estimation of heterogeneous treatment effects: From theory to learning algorithms*. in *International Conference on Artificial Intelligence and Statistics*. 2021. PMLR.

2. Künzel, S.R., et al., *Metalearners for estimating heterogeneous treatment effects using machine learning.* Proceedings of the National Academy of Sciences, 2019. **116**(10): p. 4156-4165.

3. Zhang, Y., et al., *Comparison of Prediction Models for Acute Kidney Injury Among Patients with Hepatobiliary Malignancies Based on XGBoost and LASSO-Logistic Algorithms.* International journal of general medicine, 2021. **14**: p. 1325-1335.

4. Chen, Z., H. Luo, and L. Xu, *Machine learning models of ischemia/hemorrhage in moyamoya disease and analysis of its risk factors.* Clinical Neurology and Neurosurgery, 2021. **209**: p. 106919.

5. Hou, N., et al., *Predicting 30-days mortality for MIMIC-III patients with sepsis-3: a machine learning approach using XGboost.* Journal of Translational Medicine, 2020. **18**(1): p. 462.

6. Wei, C., et al., *Machine learning model for predicting acute kidney injury progression in critically ill patients.* BMC Medical Informatics and Decision Making, 2022. **22**(1): p. 17.

7. Setty, V.M.V.A., *XGBoost Algorithm.* Towards Data Science, 2019.

8. Chernozhukov, V.a.C., Denis and Demirer, Mert and Duflo, Esther and Hansen, Christian and Newey, Whitney and Robins, James, *Double/Debiased Machine Learning for Treatment and Causal Parameters.* arXiv, 2016.

9. Mitchell Koch, J.B., *Leveraging Causal Modeling to Get More Value from Flat Experiment Results.* doordash engineering, 2020.
